# Purinergic Receptors in Neurological Diseases With Motor Symptoms: Targets for Therapy

**DOI:** 10.3389/fphar.2018.00325

**Published:** 2018-04-10

**Authors:** Ágatha Oliveira-Giacomelli, Yahaira Naaldijk, Laura Sardá-Arroyo, Maria C. B. Gonçalves, Juliana Corrêa-Velloso, Micheli M. Pillat, Héllio D. N. de Souza, Henning Ulrich

**Affiliations:** ^1^Department of Biochemistry, Institute of Chemistry, University of São Paulo, São Paulo, Brazil; ^2^Department of Neurology and Neuroscience, Medical School, Federal University of São Paulo, São Paulo, Brazil

**Keywords:** Parkinson's disease, amyotrophic lateral sclerosis (ALS), multiple sclerosis, neurodegeneration, ataxia, Huntington's disease, restless leg syndrome, purinergic receptors

## Abstract

Since proving adenosine triphosphate (ATP) functions as a neurotransmitter in neuron/glia interactions, the purinergic system has been more intensely studied within the scope of the central nervous system. In neurological disorders with associated motor symptoms, including Parkinson's disease (PD), motor neuron diseases (MND), multiple sclerosis (MS), amyotrophic lateral sclerosis (ALS), Huntington's Disease (HD), restless leg syndrome (RLS), and ataxias, alterations in purinergic receptor expression and activity have been noted, indicating a potential role for this system in disease etiology and progression. In neurodegenerative conditions, neural cell death provokes extensive ATP release and alters calcium signaling through purinergic receptor modulation. Consequently, neuroinflammatory responses, excitotoxicity and apoptosis are directly or indirectly induced. This review analyzes currently available data, which suggests involvement of the purinergic system in neuro-associated motor dysfunctions and underlying mechanisms. Possible targets for pharmacological interventions are also discussed.

## Introduction

The unexpected discovery and description of non-adrenergic and non-cholinergic inhibitory nerves working through adenosine triphosphate (ATP) and its metabolites gave rise to the introduction of the purinergic system concept in the early 70's (Burnstock et al., 1970; Burnstock, 1972). Later, purines were also described as important co-transmitters in both central (CNS) and peripheral nervous systems, as they are able to modulate and be modulated by many other neurotransmission systems and signaling pathways (Burnstock, 1997, 2009; Abbracchio et al., 2009).

After proposal of purinergic neurotransmission, the following decades were dedicated to the isolation and characterization of the two families of purinergic receptors, which are distinguished by their main agonists: P1 receptors, a family of protein G-coupled metabotropic adenosine (A_1_, _2A_A_2A_, A_2B_, A_3_) receptors, and P2 receptors. P2 receptors are sub-divided into P2X(1–7) channels, activated by ATP, and G protein-coupled metabotropic P2Y(1–12) receptors, which show sensitivity to ATP, adenosine diphosphate (ADP), uridine di- and triphosphate (UDP and UTP, respectively), or UDP-glucose depending on the receptor subtype. Beyond receptors, membrane nucleotide/nucleoside transporters and channels (e.g., pannexins) as well as ectonucleotidases play important roles in purinergic signaling. These are responsible for the exchange of purines between intracellular and extracellular environments and their enzymatic extracellular conversion, respectively (Zimmermann et al., 1998; Zimmermann, 2006; Scemes et al., 2007; Abbracchio et al., 2009; Lapato and Tiwari-Woodruff, 2017).

P2X receptors are ion channels that promote a non-selective exchange of cations, mainly Ca^2+^, Na^+^, Mg^2+^, and K^+^. ATP-activation of P2X receptors is especially important for Ca^2+^-induced intracellular signaling pathways (Surprenant and North, 2009; Puchałowicz et al., 2015). P2Y and adenosine receptors are coupled to Gq/Gi/Gs proteins, depending on the receptor subtype (Puchałowicz et al., 2015). The activation of Gq proteins triggers a signaling cascade through phospholipase C/inositol-1,4,5-triphosphate (PLC/IP3), resulting in the release of Ca^2+^ from the endoplasmic reticulum into the cytoplasm. Gs/Gi protein activation, however, will work through the stimulation/inhibition of adenylate cyclase, respectively, with subsequent up- or down-regulation of cyclic AMP (cAMP) production. Final effects of purinergic receptor-promoted signaling will depend on the cell type and other intra-/inter-cellular conditions, as i.e., in physiological embryonic and adult neurogenesis (Oliveira et al., 2016), and in various pathological scenarios, such as inflammatory (Beamer et al., 2016; Madeira et al., 2017; Przybyła et al., 2018), oncological (Allard et al., 2016; Vijayan et al., 2017; Whiteside, 2017; Kazemi et al., 2018), neurological (Burnstock et al., 2011; Stockwell et al., 2017), metabolic (Lindberg et al., 2015; Csóka et al., 2017; Parpura et al., 2017; Tozzi and Novak, 2017; Labazi et al., 2018), psychiatric (Cunha, 2008; Lindberg et al., 2015; Ortiz et al., 2015; Krügel, 2016; Cheffer et al., 2017; Oliveros et al., 2017), cognitive (Illes and Verkhratsky, 2016), and peripheral neuromuscular and/or neuromotor diseases (Robitaille, 1995; Kalmar, 2005; Burnstock et al., 2013; Jiménez et al., 2014; Bogacheva and Balezina, 2015; Puchałowicz et al., 2015; Safarzadeh et al., 2016).

In the CNS, extracellular nucleotides also participate as messengers for communication between neuronal and non-neuronal cells. As key players in neuron-glia interactions and microglial activation (Fields and Burnstock, 2006; Cunha, 2008, 2016; Färber et al., 2008; Boison et al., 2010; Lecca et al., 2012; Tsuda and Inoue, 2016; Inoue, 2017; Tsuda, 2017), both adenosine and ATP are essential modulators of neuroinflammatory responses, excitotoxicity, oxidative stress and cell death, especially via A_2A_ and P2X7 receptors activity, respectively (Cunha, 2016; Borea et al., 2017; Faas et al., 2017; Faria et al., 2017; He et al., 2017; Lu et al., 2017; Miras-Portugal et al., 2017; Vuorimaa et al., 2017). Differently from other P2X receptors, the P2X7 receptor subtype needs higher ATP concentrations for channel opening and Ca^2+^ influx and remains longer activated, recruiting pannexin pores (Volont et al., 2012; Sun et al., 2013). Through pannexin pores, large amounts of ATP are released into the extracellular environment, stimulating other purinergic receptors, and signaling cascades widely associated with pathological conditions (Bartlett et al., 2014), such as the A_2A_ receptor, which is activated by adenosine released from damaged cells or produced from ATP hydrolysis (Cunha, 2016).

**Figure 1:**
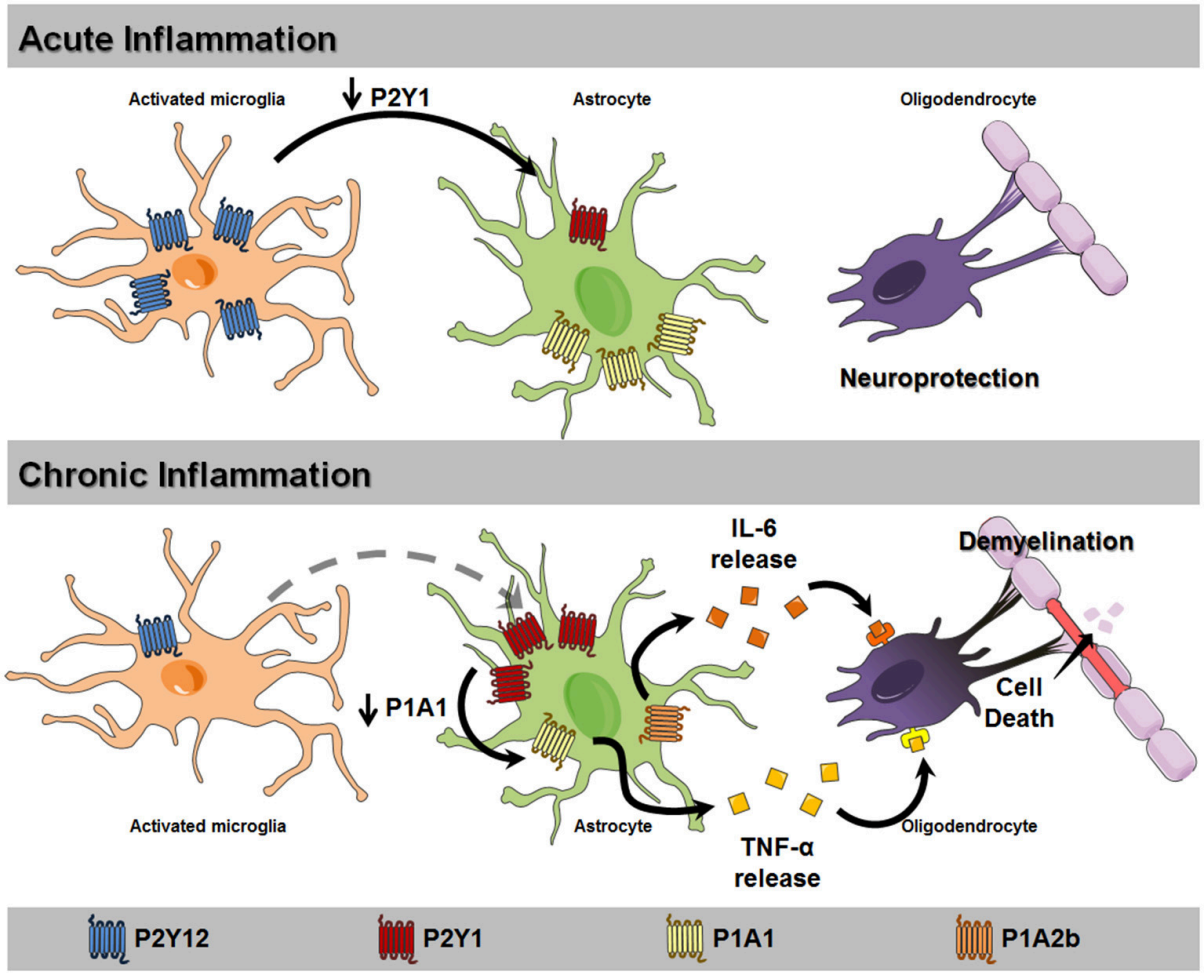
Proposed mechanism for glial purinergic dysfunction leading to loss of myelination and cell death. In acute inflammation scenarios, microglial activation upregulates P2Y12 receptor expression and activity (blue), stimulating microglial motility to the injury site. The activation of these receptors reduces P2Y1 receptor (red) expression in astrocytes, increasing reactive astrogliosis and promoting neuroprotection. Chronic inflammation, as observed in motor neuron diseases (MND), unable of upregulating microglial P2Y12 receptor expression results in constant astrocytic P2Y1 receptor activation and reduction of A_1_ receptor expression (yellow). These events result in stimulating tumor necrosis factor α (TNF-α) release, which in turn induces A_2B_ receptor activation (orange) and release of IL-6. These detrimental factors induce oligodendrocyte death and neuron demyelination, aggravating the pathological scenario.

Here, we explore the importance of purinergic signaling in neurological diseases with motor symptoms, including Parkinson's disease (PD), motor neuron diseases (MND), multiple sclerosis (MS), amyotrophic lateral sclerosis (ALS), Huntington's Disease (HD), restless leg syndrome (RLS), and ataxias. We discuss common mechanisms already known to be involved in these conditions (Table 1), revise the role of the purinergic system in demyelination processes (Figure 1), and discuss new insights for further neural pathologies that might have motor impairments to identify potential targets for pharmacological therapies to decelerate disease progression and improve motor activity.

**Table 1:** Evidence of purinergic receptors involvement in neurological diseases with major motor dysfunctions.

**Disease**	**Purinergic involvement**	**Model/Sample**	**Drug**	**Effects**	**References**
ALS	P2X4 receptor positive activity modulation	MNs culture/SOD1 (G93A) mice	Preincubation with Ivermectin (10 mM);	Neuroprotective against glutamate-induced excitotoxicity;	Andries et al., 2007
			Ivermectin 12 mg per liter of water during 70 days	Improves lifespan and increases ventral horn MNs numbers	
	P2X4 receptor	SOD1 (G93A) rats	–	Strong immunoreactivity in the ventral horns	Casanovas et al., 2008
	P2X7 receptor activation	Microglial cells derived from transgenic SOD1 (G93A) mice;	BzATP (10 and 100 μM)	Increase in NOX2 activity and ROS synthesis	Apolloni et al., 2013b
			BzATP (10 μM)	Transition from microglial M2 to M1 activated phenotype, increased TNF-α production and COX2 activation	D'Ambrosi et al., 2009
		Microglia/Neuron co-culture	BzATP (10 μM)	Cell death due to ROS and NOS production	Skaper et al., 2006; D'Ambrosi et al., 2009
		Cultured rat spinal cord MNs	ATP (1–100 μM)	MNs cell death through peroxinitrite/Fas death pathway	Gandelman et al., 2013
		MNs co-cultured with SOD1G93 astrocytes	ATP (100 μM, 5 days) or BzATP (10 μM, 48 h)	Astrocytes become neurotoxic for MNs through increased oxidative stress	Gandelman et al., 2010
	P2X7 receptor deletion	P2X7^(−/−)^/SOD1-G93A mice	–	Accelerates disease onset and progression, increased pro-inflammatory markers as well as astrogliosis, microgliosis, and MNs cell death	Apolloni et al., 2013a
	A_2A_ receptor antagonism	SOD1 (G93A) mice	Caffeine, 1.5 mg/day for 70 days, in drinking water	Shortened mice survival	Potenza et al., 2013
		Rat spinal cord cells culture	Chronic enprofylline treatment	Decreased MNs susceptibility to excitotoxic environment through inhibition of BDNF-promoted death pathway	Mojsilovic-Petrovic et al., 2006
	A_2A_ receptor expression or levels	SOD1 G93A mice	–	Decreased expression in spinal cord	Potenza et al., 2013
		SOD1 G93A mice and end-stage humans with ALS	–	Increased expression in spinal cord of symptomatic mice and patients	Ng et al., 2015
		ALS patient lymphocytes	–	Increased density in lymphocytes, positively related with clinical status of patients	Vincenzi et al., 2013
	A_2A_ receptor activation	SOD1 G93A mice	CGS21680, 5 mg/kg, i.p., during 4 weeks	Delays ALS onset possibly by stimulating non-truncated forms of the TrkB receptor	Yanpallewar et al., 2012
		NSC34 cells	T1–11 (30 μM)	Normalized abnormal cellular redistribution of human antigen R, found in MNs of ALS patients	Liu et al., 2015
	A_1_ and A_2A_ receptors	SOD1 G93A mice presymptomatic (4–6 weeks old) symptomatic (12–14 weeks old)	N(6)-cyclopentyladenosine (50 nM); CGS 21680 (5 nM)	Impaired cross-talk between receptors in presymptomatic mice, increased A_1_ receptor activation in symptomatic mice	Nascimento et al., 2015
Spinal muscular atrophy	ATP response	iPSC-derived astrocyte culture from SMA patients	ATP (10 μM)	Increased basal intracellular calcium levels accompanied by a reduced calcium response to ATP application	McGivern et al., 2013
Multiple sclerosis	P2X7 receptor expression and protein levels	Cultured PBMC from MS patients	Glatiramer acetate (50 μg/ml, 48 h) BzATP (300 μM, 30 min)	Glatiramer acetate, used to treat MS patients, reduced P2X7 receptor expression in BzATP-stimulated cells	Caragnano et al., 2012
		MS patients' spinal cords	–	Increased P2X7 receptor protein levels in microglia	Yiangou et al., 2006
		EAE rat brains	–	Increased P2X7 receptor expression related to synaptosomal fraction in the symptomatic phase and to the glial fraction in recovered rat brains	Grygorowicz et al., 2011
	P2X7 receptor polymorphisms	MS patients	–	Patients with T allele of rs17525809 polymorphism present a more prominent activity, which may contribute to MS development	Oyanguren-Desez et al., 2011
	P2X7 receptor deletion	P2X7R^−/−^ EAE mice model	–	Enhanced mouse susceptibility to EAE	Chen and Brosnan, 2006
				Suppressed clinical symptoms in EAE mice	Sharp et al., 2008
	P2X7 receptor antagonism	EAE mouse model	BBG (10 mg/kg daily, delivered from pellets, during 20 days)	Antagonism improved symptoms and promoted remyelination	Matute et al., 2007
	P2Y12 receptor levels	MS patients cortical tissue	–	Reduced protein levels near demyelination areas	Amadio et al., 2010
	P2Y12 receptor deletion	P2Y12 knockout EAE mice	–	Mice developed more severe EAE related to higher release of IL-23 cytokines and imbalanced Th-cell subtype frequencies	Zhang et al., 2017
	A_2A_ receptor antagonism	MS patients	Coffee consumption exceeding 900 mL daily	Reduced MS risk in comparison to control group	Hedström et al., 2016
	A_1_ receptor deletion	A1AR^−/−^ EAE mice		Induced severe EAE, with more prominent demyelination, axonal injury, and microglia activation	Tsutsui et al., 2004
	A_1_ receptor activation	A1AR^−/−^ EAE mice	Caffeine (2 mg/kg) + adenosine amine congener (10 μg/kg), subcutaneous pump, during 25 days	Reduced EAE severity induced by A_1_ receptor expression deletion	Tsutsui et al., 2004
		Cultured PBMC from MS patients	R-phenylisopropyl-adenosine (1 mM)	Inhibited IL-6 production	Mayne et al., 1999
Parkinson's disease	P2X1 receptor antagonism	H4 cells overexpressing α-synuclein	Pre-treatment with NF449 (1–5 μM) followed by 48 h treatment with ATP (3 mM, every hour)	Prevented ATP-induced α-synuclein aggregation in a dose dependent manner	Gan et al., 2015
	P2X7 receptor antagonism	6-OHDA lesioned rats	A-438079 (30 mg/kg, i.p., before lesion establishment)	Prevented depletion of dopamine in striatum without reducing dopaminergic neuron cell death	Marcellino et al., 2010
			BBG (45 mg/kg, i.p., every 48 h during 2 weeks, before lesion establishment)	Prevented loss of tyrosine-hydroxylase immunoreactivity and attenuated rotational behavior and memory deficit	Carmo et al., 2014
			BBG (50 mg/kg, i.p., daily, during 1 week, after lesion establishment)	Reverted dopaminergic neurons loss in substantia nigra and rotational behavior	Ferrazoli et al., 2017
		BV2 microglia cells	Pretreatment with BBG (1 μM)	Antagonism and/or deletion of P2X7 receptor blocked the interaction between α-synuclein and P2X7 receptors and decreased ROS production induced by α-synuclein	Jiang et al., 2015
		SH-SY5Y cells	Pretreatment with PPADS (100 μM) or AZ 11645373 (10 μM)	Prevented abnormal calcium influx induced by α-synuclein	Wilkaniec et al., 2017
	P2X7 receptor polymorphism	PBMC from PD patients	–	1513A>C (rs3751143) polymorphism increased PD risk by facilitating pore formation and cell death	Liu et al., 2013
	P2Y6 receptor antagonism	SH-SY5Y cells	Pretreatment with MRS2578 (1.0 μM)	Decreased ROS production and other inflammatory markers induced by MPP+	Qian et al., 2017
	A_2A_ receptor antagonism	6-OHDA lesioned rats	8-ethoxy-9-ethyladenine (8 mg/kg, daily, during 28 days, minipumps)	Enhanced effect of low doses of L-DOPA without increased dyskinesia	Fuzzati-Armentero et al., 2015
		MPTP treated monkeys	KW-6002 (10.0 mg/kg, orally)	Increased effect of D2 receptor agonist quinpirole, D1 receptor agonist SKF80723 and low doses of L-DOPA without increased dyskinesia	Kanda et al., 2000
		PD patients with PD gene risk variant *LRRK2* R1628P	Caffeine intake through coffee and tea consumption	Decreased PD risk in subjects with LRRK2 variant R1628P	Kumar et al., 2015
		PD patients with GRIN2A variant rs4998386-T allele	Caffeine intake through coffee consumption	Increased protective effect of GRIN2A variant rs4998386-T allele	Hamza et al., 2011; Yamada-Fowler et al., 2014
		A_2A_ receptor knockout mice, SH-SY5Y cells	SCH 58261, ZM 241385	Decreased α-synuclein aggregation, prevent neuronal death induced by extracellular α-synuclein and restrain overactivation of NMDA receptors	Ferreira et al., 2015
		Brain slices from mice treated with MPTP	Preladenant (5 μM)	Facilitated beneficial microglial responses to injury	Gyoneva et al., 2014
		Rats treated with LPS	Caffeine 10 and 20 mg/kg; KW6002 1.5 and 3 mg/kg; i.p. for 6 days	Prevented striatal dopaminergic deficit and hydroxyl radicals release	Gołembiowska et al., 2013
	A_2A_ receptor number	Mice injected with α-Syn fibrils	–	Hippocampal A_2A_ receptors number increased after injections of α-synuclein in mice	Hu et al., 2016
	A_2A_ receptor polymorphisms	PD patients	–	rs3032740 and rs5996696 polymorphisms are inversely linked to PD risk	Popat et al., 2011
Huntington's disease	P2X7 receptor antagonist	Tet/HD94 and R6/1	BBG (45.5 mg/kg, i.p., every 48 h during 28 days)	Reduce body weight loss, improve motor functions, and prevent neuronal loss	Diaz-Hernandez et al., 2009
	A_1_ receptor agonist	3-NPA mouse and rat model	Pre-treatment of R-PIA (1.75 mg/kg, i.p.) 15 min prior 3-NPA application	Reduction of seizure but not prevention of neuronal loss	Zuchora and Urbañska, 2001
		3-NPA rat model	ADAC (100 μg/kg, i.p., daily for 2 days) 3 days after 3-NPA	Reduction in striatal lesion and degeneration, improvement of motor functions	Blum et al., 2002
	A_1_ receptor antagonist	Intracraneal application malonate 6 μmol in Swiss-Webster mice and 3 μmol Sprague Dawley rats	Pre-treatment with CPX 1 mg/kg, i.p.	Stimulate DAergic and GABAergic neuron death	Alfinito et al., 2003
	A_2A_ receptor polymorphisms	1876 C/T		Silent mutation in A_2A_ receptor	Dhaenens et al., 2009
		1876 T/T		Accelerates HD onset by 3.5 years	
		rs2298383		Early onset of HD	Taherzadeh-Fard et al., 2010
	A_2A_ receptor antagonist	Intracranial application malonate 6 μmol in Swiss-Webster mice and 3 μmol Sprague Dawley rats	Pre-treatment with DMPX 5 mg/kg, i.p.	Provided protection to DAergic and GABAergic cells against malonate	Alfinito et al., 2003
		Human	<190 mg/day caffeine	Accelerates HD onset.	Simonin et al., 2013
		3-NPA mouse model	8-(3-chlorostyryl) caffeine (5 mg/kg and 20 mg/kg, i.p.) 2x day for 5 days prior 3-NPA application	Reduction in striatal damage	Fink et al., 2004
		R6/2 mice	SCH58261 (0.01 mg/kg, i.p.)	Reduction in striatal BDNF levels at earlier HD stage	Potenza et al., 2007
			SCH58261 (50 nM): microdialysis application in striatum)	Reduction of glutamate and adenosine level	Gianfriddo et al., 2004
			Application of SCH58261 (0.01 mg/kg, i.p.) daily for 7 days at age of 5 weeks	Reduced NMDA-induced toxicity and emotional responses	Domenici et al., 2007
		Corticostriatal slices from R6/2 mice	ZM241385 (100 nM)	Prevention of BDNF positive effect on NMDA toxicity	Martire et al., 2010
		ST14/SQ120 cells			
		Primary rat striatal culture	Pre-treatment with SCH 58261 (30 nM) prior bath application QA 900 μM	Enhanced QA-induced increase in intracellular calcium concentration	Popoli et al., 2002
		QA rat model	Pre-treatment with SCH 58261 (0.01 mg/kg, i.p.) prior to QA application	Blocked the effect of QA on striatal gliosis, EEG changes, motor activity and glutamate levels	Popoli et al., 2002
			DMPX (0.2 μg, i.p.) application 5 min after QA application	Blocked QA-induced EEG abnormalities in frontal cortex	Reggio et al., 1999
			Pre-treatment with SCH58261 (0.01 mg/kg, i.p.) 20 min before QA application	Reduction in rearing behavior and anxiety levels	Scattoni et al., 2007
			SCH58261 (0.01 and 1 mg/kg, i.p.) daily for 1 or 3 weeks	Reduction in striatal BDNF levels	Potenza et al., 2007
		Transgenic HD rat model	KW-6002 (1 and 3 mg/kg, i.p.)	No beneficial locomotor activity at 6 and 12 month age	Orrú et al., 2011
			SCH 442416 (0.3 and 1 mg/kg, i.p.)	No significant effect in reducing electromyography responses	
	A_2A_ receptor agonist	Primary rat striatal culture	Pre-treatment with CGS21680 (100 nM.) prior bath application QA 900 μM	Reduced QA-induced increase in intracellular calcium concentration	Popoli et al., 2002
		Corticostriatal slices from R6/2 mice	CGS21680 (30 nM)	Beneficial effect against NDMA-induced toxicity	Ferrante et al., 2010
		R6/2 mice	CGS21680 (5 μg/kg, i.p.) daily for 2 weeks	Delay decline in motor performance and inhibit reduction in brain weight	Chou et al., 2005
			CGS21680 (0.5 mg/kg, i.p.) daily for 3 weeks	Brain region dependent alteration in NMDA glutamate receptor subunits density	Ferrante et al., 2010
			CGS21680 (0.5 mg/kg, i.p.)	No changes in behavior compared to wild type	Martire et al., 2007
		Corticostriatal slices from R6/2 mice	CGS21680 (5 μg/kg, i.p.) daily for 2 weeks	Brain region dependent alteration in NMDA subunits	Ferrante et al., 2010
	A_2A_ receptor knockout	N171-82Q mouse model	–	Aggravate survival and motor functions and decrease in specific markers for sub-population medium spiny neurons	Mievis et al., 2011
		3-NPA mouse model	A_2A_ receptor knockout mice treated with 3-NPA	Reduction in striatal damage	Fink et al., 2004
Ataxia	A_2A_ receptor antagonism	SCA3 mice model	Caffeine (1 g/L, drinking water during 2 weeks)	Decreased synaptotoxicity and reactive gliosis	Gonçalves et al., 2013
		(TgMJD) mice	Caffeine (1 g/L, drinking water during 2 weeks)	Prevented motor symptoms and cognitive impairment	Gonçalves et al., 2013
	P2X receptors	CHO-K1 cells with mutant PKCγ	ATP (1 mM)	Increased damaging aggregation of mutant PKCγ	Seki et al., 2005
Restless leg syndrome	A_2A_ receptor	Iron deficient mice	–	Increased in striatal presynaptic neurons	Gulyani et al., 2009
	A_1_ and A_2A_ receptors	Iron deficient mice	–	Decreased A_1_ and D2 receptor density in animals with mild, moderate and severe deficiency; increased pre-synaptic A_2A_ receptor density in the latter	Quiroz et al., 2016

## Motor neuron diseases

Motor neurons (MNs) are classified in different categories according to their soma location, electrical speed transmission, and other cellular and physiological characteristics. Regarding the location of their somas, MNs can be classified as upper or lower MNs. Lower MNs have their soma located either in the brainstem—where they control head and neck muscle contraction through cranial nerves—and in the anterior horn of the spinal cord—where their axons innervate and control skeletal muscle contraction through spinal nerves. Upper MNs have somas located in the primary motor cortex and axons that project either to the brainstem or to the spinal cord through corticobulbar and corticospinal tracts, respectively. Upper MNs axons in the brainstem interact with lower MNs, regulating their control of head and neck contraction, while upper MNs project to the spinal cord synapse with lower MNs that innervate skeletal muscles, controlling their contraction (Rezania and Roos, 2013; Verschueren, 2017).

MND are neurodegenerative conditions that affect MNs and result in motor dysfunctions without compromising sensorial neurons. MND are classified according to the damage location in relation to the spinal cord. Diseases affecting lower MNs and upper MNs are known as lower MNDs and upper MNDs, respectively. Lower MNDs include progressive muscular atrophy, spinal muscular atrophy (SMA), spinal and bulbar muscular atrophy, and monomelic amyotrophy (Hirayama disease). However, the most prevalent subtype of MND is ALS, in which both upper and lower MNs are affected and where non-neuronal cells as microglia and astrocytes play a central role in its pathogenesis and progression (Rezania and Roos, 2013; Verschueren, 2017).

### Amyotrophic lateral sclerosis

ALS is the main motor disorder in adulthood. It is characterized by a progressive loss of MNs from the motor cortex, brainstem, and spinal cord (Kiernan et al., 2011). As a result of this neuronal loss, muscle weakness, spasticity, and muscle atrophy occur, inducing progressive paralysis. ALS is a very aggressive pathology that usually evolves in a fast-progressive way. Patients have a lifespan of 2–5 years after diagnosis. Death is frequently due to breathing failure. Most of ALS cases (90%) are sporadic, while a small proportion (10%) is linked to genetic mutations that usually follow an autosomal dominant transmission (Harms and Baloh, 2013; Renton et al., 2013). Cognitive impairment is also associated with ALS. In fact, 30% of ALS cases develop frontotemporal dementia (Lomen-Hoerth, 2011). The C9ORF72 mutation is responsible for the main part of ALS and frontotemporal dementia inherited cases (DeJesus-Hernandez et al., 2011).

The cause of and reasons for MNs death are still unknown. Particularly, it is still not known why this specific neuronal population is affected. However, intense research performed throughout the last two decades has uncovered several hallmarks and molecular mechanisms involved in ALS neurodegeneration. Among them, neuroinflammation, which is understood to be a maintained immune system response in the CNS, plays a central role in the pathogenesis of ALS. This response includes astrocytic and microglial activation and lymphocyte infiltration (Barbeito et al., 2010). There is strong evidence for a compromised energetic metabolism in ALS. Several genes involved in the mitochondrial electron transport chain are altered in their mRNA expression levels (Ferraiuolo et al., 2007, 2011; Lederer et al., 2007; Raman et al., 2015). Further, numerous studies reported structural and functional abnormalities in mitochondria, resulting in increased reactive oxygen species (ROS) and decreased ATP production (Jung et al., 2002; Mattiazzi et al., 2002; Menzies et al., 2002; Wiedemann et al., 2002; Browne et al., 2006), with supposed impacts on purinergic signaling. Here, we will discuss the contribution of purinergic signaling in ALS etiology.

#### Purinergic involvement in ALS

##### P2X receptors

ATP mediates intercellular communication by acting as a messenger between neurons and glia via activation of several purinergic P2 receptors. The involvement of purinergic receptors in ALS has been documented, such as the P2X4 receptor subtype, which is implicated in neuroprotection (Andries et al., 2007) and microglial activation (Tsuda et al., 2003). Positive allosteric modulation of P2X4 receptor activity with Ivermectin and pre-incubation with low ATP concentration has shown to induce neuroprotection against glutamate-induced excitotoxicity in MNs cultures, a phenotype observed in several ALS models. Allosteric P2X4 receptor activation also improved the lifespan of superoxide dismutase 1 (SOD1) transgenic mice harboring the G92A mutation (Gly-93 to Ala)—a conventional animal model of ALS—by 10% and increased the number of ventral horn MNs in the spinal cord (Andries et al., 2007). Ventral horns are the main neurodegenerative regions affected in ALS. These findings indicate that purinergic receptors modulate excitability, exerting neuroprotection in ALS (Miles et al., 2002). However, it has been described that the allosteric P2X4 receptor activator Ivermectin acts on AMPA receptors inhibiting glutamate excitotoxicity, which could be also responsible for these observed beneficial effects.

Interestingly, the P2X4 receptor has been suggested as a novel marker for non-typical apoptotic and degenerating MNs both in the spinal cord and in other degenerated areas, which had not been previously linked to ALS. P2X4 receptor-immunoreactivity was enhanced in the ventral horns of SOD1 (G93A) transgenic rats. These P2X4 receptor-positive cells were surrounded by microglia with a neuronophagic phenotype (Casanovas et al., 2008). Moreover, Tsuda et al. proved that the P2X4 receptor is expressed selectively in activated microglia after neural injury in the spinal cord and that this expression is required for neuropathic pain (Tsuda et al., 2003). The same study showed that pharmacological inhibition of P2X4 receptors induced a reduction in neuropathic pain, indicating a direct relationship between P2X4 receptor activation and microglial reactivity (Tsuda et al., 2003). Further studies regarding the role of P2X4 receptors in activated and resting microglia are needed for elucidating the participation of the P2X4 receptor in ALS etiology and progression.

The P2X7 receptor is expressed in microglia (Ferrari et al., 1996), spinal cord neurons (Deuchars et al., 2001; Wang et al., 2004), astrocytes (Ballerini et al., 1996), and oligodendrocytes (Matute et al., 2007). Activated microglia from the dorsolateral white matter in the spinal cord of sporadic ALS patients presented increased P2X7 receptor immunoreactivity (Yiangou et al., 2006). This receptor has been tightly linked to neuroinflammation. *In vitro* studies also showed increased densities of P2X7 and P2X4 receptors, upregulation of P2Y6 receptor expression, and decreased ectonucleotidase CD39 hydrolytic activity in transgenic mice SOD1 (G93A)-derived microglia, all indicating a potentiation of the purinergic system in ALS. In fact, SOD1 (G93A) microglia treated with ATP or 2′-3′-O-(benzoyl-benzoyl) ATP (BzATP), a potent P2X7 receptor agonist, presented a prominent transition from the microglial M2 to the M1 activated phenotype, accompanied by augmented production of tumor necrosis factor alpha (TNF-α) and cycloxygenase 2 (COX2) (D'Ambrosi et al., 2009). BzATP treatment of SOD1 (G93A) microglia also increased the presence of inflammatory markers, such as nicotinamide adenine dinucleotide phosphate-oxidase 2 (NOX2) activity and ROS production, indicating damaging effects resulting from P2X7 receptor activation (Apolloni et al., 2013b). As expected, P2X7 receptor activation in microglia-neuronal co-culture induced cell death by ROS and reactive nitrogen species generation (Skaper et al., 2006; D'Ambrosi et al., 2009). Complementary to the involvement of P2X7 receptors, Parisi et al. (2013, 2016) reported an overproduction of several microRNAs in neuroinflammation. In agreement, expression rates of these microRNAs were upregulated in ALS models upon P2X7 receptor stimulation (Parisi et al., 2016).

Astrocytes, the most abundant cell type in the CNS, show low expression of P2X7 receptor under physiological conditions. However, this potential cytotoxic receptor presents upregulated expression and increased activity following injury or under pro-inflammatory conditions (Franke et al., 2004; Narcisse et al., 2005; Lovatt et al., 2007). SOD1 (G93A) mice-derived astrocytes showed increased extracellular ATP-induced signaling as well as increased ATP hydrolysis (Gandelman et al., 2010). As previously reported for microglia, P2X7 receptor activation resulted in astrocyte cytotoxicity accompanied by production of reactive oxygen and nitrogen species that are harmful to MNs (D'Ambrosi et al., 2009).

*In vitro* studies presented consistent data regarding P2X7 receptor function in inflammation through microglia and astrocytes, which are detrimental for MNs survival. Low doses of ATP or BzATP induced spinal MNs death through the peroxinitrite/Fas pathway (Gandelman et al., 2013). However, *in vitro* studies fail to mimic the biological interplay between neuronal and glial cell types. Activation of the Fas pathway, or “Fas-death pathway,” is required for inducing death of MNs in trophic factor deprivation environment (Raoul et al., 1999; Barthélémy et al., 2004). Fas can trigger two different signaling pathways: (1) activation of Fas-associated death domain (FADD) and caspase 8, inducing mitochondrial cytochrome c release, or (2) activation of FADD-associated protein 6 (Daxx), activating Ask1 and p38, ultimately increasing production of nitric oxide and peroxynitrite through NOS1 (Estévez et al., 1998, 2000; Raoul et al., 1999, 2002). Although the latter pathway has been described in MNs, it is not restricted to this neuronal population alone.

Though studies have linked the P2X7 receptor to neuroinflammation, surprising results have been found in ALS murine models lacking P2X7 receptor. The genetic deletion of P2X7 receptor expression (P2X7^−/−^) accelerated disease onset and progression, induced neuroinflammatory responses, and produced MNs depletion at end stages of the disease in comparison with P2X7^+/+^/SOD1 (G93A) animals (Apolloni et al., 2013a). The heterozygous SOD1 (G93A) P2X7 receptor^+/−^ animal model did not present any significant differences in body weight, disease onset or motor performance.

While the heterozygous SOD1 (G93A) P2X7 receptor^+/−^ animal model did not present any significant differences in body weight, disease onset, or motor performance, the genetic deletion of P2X7 receptor expression (P2X7^−/−^), instead of improving ALS disease conditions, accelerated disease onset and progression, induced neuroinflammatory responses, and produced MNs depletion at end stages of the disease in comparison with P2X7^+/+^/SOD1 (G93A) animals (Apolloni et al., 2013a). These detrimental effects on P2X7 receptor-knockout SOD1 (G93A) mice shed light on possible dual effects of the P2X7 receptor in maintaining normal glial activation/trophic phenotypes at early stages of ALS and promoting a pronounced immunoinflammatory response in advanced stages of the disease. Moreover, P2X7 as well as P2X4 receptor expression levels were upregulated in neurons of asymptomatic SOD1 (G93A) mouse peripheral nervous system; however, more information about the mechanisms of action of these receptors in ALS is required (Volont et al., 2016).

The P2X7 receptor has been implicated in detrimental processes other than neuroinflammation. For instance, heat shock proteins that are elements involved in the unfolded protein response are also a neuroprotective mechanism against unfolded proteins that accumulate in the endoplasmic reticulum in response to stress, a phenotype associated with several ALS models. Specifically, the heat shock protein 90 (Hsp90) expression is upregulated in SOD1 (G93A) animal models as well as in ALS patients. However, it is not clear whether this upregulation is beneficial or prejudicial as *in vitro* studies reported that Hsp90 is able to induce MNs cell death through P2X7 receptor and FAS signaling (Franco et al., 2013). On the other hand, two chaperones, HSP90α and HSP70-1A, interact with A_2A_ purinergic receptors. By this interaction, they retain the receptor in the endoplasmic reticulum prior to exportation, ensuring its correct folding and acting as a protein quality control system (Bergmayr et al., 2013).

##### P2Y receptors

The metabotropic P2Y12 receptor has been proposed as a marker for ALS progression. It is co-expressed with CD11b in microglia and is also functional in oligodendrocytes. Its immunoreactivity is gradually lost in the dorsal and ventral horns of the spinal cord during ALS disease in the SOD1 (G93A) model, while CD68 immunoreactivity increases, indicating that P2Y12 receptor expression as marker for M2 microglia (Amadio et al., 2014). However, no specific function of this receptor has yet been described in association with either microglia or oligodendrocytes.

##### Adenosine receptors

Among the four adenosine receptors, the A_2A_ receptor subtype has been mostly described to be involved in ALS. *In vivo* and *in vitro* studies suggest a role of A_2A_ receptor associated with both improvement and attenuation of ALS progression, which could suggest a stage-dependent role of this receptor.

The A_2A_ receptor has been reported as the main target for caffeine, a non-selective adenosine antagonist (Fredholm et al., 1999; Karcz-Kubicha et al., 2003). The first investigation of the possible neuroprotective effect of caffeine intake and ALS development was performed in an epidemiological study, showing a reduced ALS risk in 377 European patients (Beghi et al., 2011). However, a longitudinal analysis based on over one million individuals from five cohort studies failed to demonstrate this association (Fondell et al., 2015). Similarly, an Italian case-control study found no association with caffeine intake (Pupillo et al., 2017). In the SOD1 (G93A) ALS mouse model, A_2A_ receptor blockade by chronic consumption of caffeine shortened survival and decreased motor performance (Potenza et al., 2013). An interesting finding of this study was the decrease in A_2A_ receptor protein levels only in the spinal cord from the SOD1 (G93A) control group and not in caffeine-treated animals. Whether this downregulation of receptor protein expression is due to the heterogeneity of analyzed cell types (MNs, astrocytes and microglia) or a true outcome of the disease must be determined. In fact, another study showed an increased expression of A_2A_, but not A_1_ receptors, in the spinal cords of symptomatic SOD1 (G93A) mice and in spinal cords of human end-stage ALS patients (Ng et al., 2015).

In the pathophysiology of ALS, one described mechanism that is associated with susceptibility of MNs to excitotoxic insults is activation of the receptor tropomyosin kinase receptor B (TrkB) by brain-derived neurotrophic factor (BDNF) (Fryer et al., 2001; Hu and Kalb, 2003). In this pro-death pathway, BDNF (Koh et al., 1995; Ishikawa et al., 2000; Kim, 2003) agonist stimulation of A_2A_ receptors leads to the damaging transactivation of TrkB (Lee and Chao, 2001; Rajagopal et al., 2004). This neurotoxic pathway is diminished by blockade of A_2A_ receptor in rat MNs *in vitro* injured by the levels of ALS-related mutated proteins, such as SOD1 (G85R) and p150glued (G59S) (Mojsilovic-Petrovic et al., 2006). A physical interaction between TrkB and A_2A_ receptor was demonstrated, in which their disruption by cholesterol depletion blocks the detrimental effect of BDNF to render MNs vulnerable to insult in a similar way observed by *in vitro* A_2A_ receptor blockade (Mojsilovic-Petrovic et al., 2006). In addition to pharmacological inhibition, partial genetic ablation of A_2A_ receptors in SOD1 (G93A) mice protected MNs from astrocyte-induced cell death and delayed disease progression in the mouse model (Ng et al., 2015).

During neuromuscular transmission, adenosine is an important modulator of acetylcholine release by acting on both inhibitory A_1_ and excitatory A_2A_ receptors (Correia-de-Sá et al., 1991). In pre-symptomatic SOD1 (G93A) mice, a loss of functional cross-talk between A_1_ and A_2A_ receptors was reported, suggesting adenosine signaling dysfunction prior to ALS onset (Nascimento et al., 2015). In the early asymptomatic ALS phase, activation of A_2A_ receptors by the agonist CGS21680 enhanced acetylcholine-evoked release, whereas this excitatory effect was no longer observed during the symptomatic phase (Nascimento et al., 2015). Intracellular Ca^2+^ homeostasis was also dysfunctional in MNs from SOD1 (G93A) mice (Fuchs et al., 2013). A_2A_ receptor activation increased the levels of cytosolic Ca^2+^ (Kobayashi et al., 1998; Palma et al., 2011), while the opposite effect was observed after A_2A_ receptor blockade (Li and Wong, 2000; Correia-de-Sá et al., 2002) and after A_1_ receptor activation (De Lorenzo et al., 2004). The described loss of a functional equilibrium between A_1_ and A_2A_ receptor actions in presymptomatic ALS mice could induce a hyperexcitable adenosinergic tonus in neuromuscular transmission, contributing to the Ca^2+^-mediated excitotoxicity at initial stages of the disease (Nascimento et al., 2015). According to this hypothesis, A_2A_ receptors could act in an excitatory context during the pre-symptomatic phase, whereas A_2A_ receptor excitatory action disappears during the symptomatic phase (Nascimento et al., 2015). This stage-dependent effect of A_2A_ receptors could explain the different effects on modulation of this receptor in ALS models. Nevertheless, further investigation of this receptor through ALS progression is needed.

Outside the neuromuscular context, only A_2A_ receptor density was up-regulated in lymphocytes from ALS patients, while A_1_, A_2B_, and A_3_ receptors densities and affinities did not change compared to age-matched healthy subjects. Surprisingly, A_2A_ receptor density was positively correlated with improved clinical and functional status according to the revised ALS Functional Rating Scale (Vincenzi et al., 2013). Furthermore, cAMP production in ALS lymphocytes was increased by pharmacological stimulation of the A_2A_ receptor by its agonist CGS21680. Within the immune system, higher levels of cAMP reduce the production of pro-inflammatory mediators and increase the production of anti-inflammatory factors (Raker et al., 2016). Therefore, in addition to its described anti-inflammatory function (Sitkovsky, 2003; Haskó, 2004), these findings indicate a possible protective role for the A_2A_ receptor, specifically in the peripheral immune system.

In MNs, aberrant RNA metabolism—due to mislocalization and/or dysfunction of RNA-binding proteins—has been implicated in ALS (Strong, 2010). Human antigen R, a RNA-binding protein that translocates from nucleus to the cytoplasm, could be associated with pathogenic pathways of ALS (Liu et al., 2015). Stimulation of A_2A_ receptors with the agonist T1–11 normalized the cellular redistribution of human antigen R in the MNs cell line NSC-34, providing potential therapeutic interventions for improving the sustainability of MNs against stress and delaying ALS progression.

##### Conclusion

ALS is a multifactorial disease with a marked loss of MNs and an important contribution of non-neuronal cells to its pathogenesis and progression. Several works reported the involvement of diverse elements from the purinergic system in ALS, with a critical contribution to neuroinflammation through microglia and astrocyte activation. Two elements play a crucial role in ALS pathogenesis regarding the purinergic system. P2X4 and P2X7 receptors participate in microglia reactivity and astrogliosis, which both produce detrimental effects on MNs maintenance and survival. However, their involvement in ALS progression is more complex as shown *in vivo* models. There is a specific time window, at late pre-symptomatic stages of the disease, where antagonism of the purinergic P2X7 receptor may be beneficial. However, P2X7 receptor inhibition after this point produces negative effects on cell survival. During the early phase of ALS, the A_2A_ receptor mediates excitotoxicity effects on neuromuscular junction, whereas this effect is no longer observed with the progression of the disease, at the symptomatic phase. These observations indicate a possible change of function of this receptor depending on disease state. In terms of the variety of extracellular nucleotide-degrading enzymes and purinergic receptors, which assemble as homo- or heterocomplexes and vary in composition in different CNS cell types, more intense research has to be performed to clarify short- and long-term implications of purinergic signaling in ALS.

### Other motor neuron diseases

Spinal Muscular Atrophy (SMA) is a MND that affects MNs in the spinal cord and brainstem. Patients share manifestations similar to ALS, such as weakness, muscle atrophy/paralysis, and respiratory impairment that can lead to death (Crawford and Pardo, 1996; Lefebvre et al., 1997). The most frequent type of SMA is caused by deletions in the survival motor neuron 1 (SMN1) gene, which is involved in biosynthesis of RNA and proteins (Burghes et al., 1994; Lefebvre et al., 1995; Jablonka et al., 2000; Gabanella et al., 2007; Bebee et al., 2010; Lotti et al., 2012). High SMN1 expression in neurons and glia in a SMA transgenic mice model rescued MNs survival, indicating a non-autonomous cellular contribution in SMA (Gavrilina et al., 2008).

Currently, the only study shedding light on the involvement of the purinergic system in SMA used human induced pluripotent stem cell (iPSC)-derived astrocytes (McGivern et al., 2013). Authors reported that this population presented increased basal cytosolic Ca^2+^ concentrations and reduced responses to ATP application, suggesting a possible impairment of the purinergic system in the disease. For instance, P2Y2 receptor activation triggered intracellular Ca^2+^ mobilization in control cells, which was not observed in iPSC-derived astrocytes (Zhu and Kimelberg, 2001; Verkhratsky et al., 2012). The cause for this dysfunction, either because of altered kinetics or compromise of downstream signaling elements, still needs to be clarified. Further, this work was restricted to P2Y2 receptors, while other purinergic receptor subtypes may be involved in the same pathology. The role of the purinergic system in SMA disease should also be studied in microglia and neurons for postulating mechanisms of purinergic signaling in this MNs disorder.

Although no direct experimental evidence links the purinergic system to other previously mentioned MND, the participation of P2 and A_2A_ receptors in the physiology of peripheral nervous system is well-established. Schwann cells—peripheral glial cells responsible for myelin maintenance and injury—destroy their own myelin after peripheral nerve injury and remove myelin and cell debris (Band et al., 1986). Since demyelination is a secondary process present in MND pathophysiology, Schwann cell activity may contribute to disease progression. In fact, injured sciatic nerve induces Schwann cell proliferation via ATP activation of P2X7 receptor while A_2A_ receptors activation inhibits it, both through MAPK/ERK pathways (Stevens and Fields, 2000; Song et al., 2015).

Schwann cells located near the amphibian neuromuscular junction are activated by synaptic ATP release (Robitaille, 1998), and purinergic signaling has key roles in presynaptic modulation (Todd and Robitaille, 2006). However, as suggested by the use of suramin (a non-selective P2 receptor antagonist), activation of Schwann cells by local applications of ATP did not depend on P2 receptors, indicating a possible involvement of A_1_ receptor activation by ATP metabolites (Rochon et al., 2001). An *in vitro* model of neuromuscular junction injury showed that ATP was an activating signal for Schwann cells in response to nerve function impairment, triggering purinergic signaling (Rodella et al., 2017). Xu et al. (2013) brought evidence for the involvement of purinergic signaling pathway in glia-derived neurotrophic factor (GDNF) release by Schwann cells in nerve injure. ALS patients presented increased GDNF levels in the cerebrospinal fluid in comparison to control groups as a protective response to nerve injury (Grundström et al., 2000). Moreover, ATP and ADP released by injured nerves activated purinergic receptors that stimulate protein kinase-C and -D pathways (Xu et al., 2013). Furthermore, purinergic receptors promoted myelination processes in oligodendrocytes and inhibited them in Schwann cells. The importance of the purinergic system involvement in demyelination process in MND is clear, but a better understanding of the degenerative process is necessary for developing therapies (Xu et al., 2013).

## Multiple sclerosis

MS is an autoimmune disease of the CNS. It is estimated to affect ~2.5 million people worldwide and is highly incapacitating; 50% of patients will need to use a wheelchair in the years following disease onset between 25 and 45 years of age. The symptomatology of MS is heterogeneous and includes motor impairment, cognitive, visual, and sensory deficits, fatigue, and pain (Compston and Coles, 2008). The etiology of MS is unknown. However, it is speculated that environmental and genetic factors play a role in disease development (Dendrou et al., 2015).

The pathophysiology of MS is characterized by chronic inflammation, in which T cells become responsive for different myelin epitopes, triggering a cascade of events resulting in axonal demyelination and neuronal transmission impairment (Sun et al., 1991; Koehler et al., 2002). The hallmarks of MS are axonal loss, astrogliosis/microgliosis, oligodendrocytes damage, inflammatory focal lesions and T-cell activation (Goldenberg, 2012; Luo et al., 2017). There is a range of immune modulatory drugs used to alleviate MS symptoms. However, these drugs induce troubling side effects including development of other autoimmune disorders and fatal opportunistic infections (Dendrou et al., 2015). Thus, better understanding of the disease in order to develop more effective and safe treatments is needed.

### Purinergic involvement in MS

The first report of the involvement of purinergic signaling in MS came from Mayne et al. (1999), when it was found increased plasma and serum TNF-α levels in MS patients correlated with low levels of adenosine. The induced experimental autoimmune encephalomyelitis (EAE) mouse model, established by myelin oligodendrocyte glycoprotein or myelin basic protein peptide inoculation and immunization, provides some clues on the mechanical role of purinergic signaling in early-stage MS. The EAE model shows similar features as seen in the CNS of MS patients, such as infiltrating T-cells and presence of IgG antibodies as well as hind limb paralysis (Lassmann, 1983; Miller and Karpus, 1994; Eng et al., 1996; Constantinescu et al., 2011). The four purinergic receptors especially known to be involved in MS are P2X7, P2Y12, A_1_, and A_2A_ receptors.

#### P2X receptors

The participation of P2X receptors in MS has been proposed, since they modulate astrocytes and axon-oligodendrocyte communication, which is necessary for myelination formation and repair (Butt, 2006). *Post-mortem* tissue from MS patients exhibited increased P2X7 receptor expression in microglia from spinal cord and brain white matter (Yiangou et al., 2006) in astrocytes localized in active brain lesions (Narcisse et al., 2005) and in oligodendrocytes from optic nerve samples (Matute et al., 2007). Immunohistochemistry analysis of brain sections from the frontal cortex of MS patients showed immunostaining for P2X1, P2X2, P2X3, P2X4, and P2X7 receptors, while P2X6 receptor subunits could not be detected (Amadio et al., 2010). Analysis of blood monocytes from MS patients did not show any differences in P2X7 receptor expression in comparison to healthy controls (Caragnano et al., 2012). However, monocytes from MS patients undergoing treatment with glatiramer acetate—which acts displacing myelin basic protein from the binding site on MHC-II molecules, preventing the activation of myelin-specific T cells—exhibit reduced P2X7 receptor and interleukin (IL)-1β expression, indicating that this treatment may act by decreasing P2X7 receptor pro-inflammatory effects (Caragnano et al., 2012).

Alterations in the P2X7 receptor gene have been identified in MS, leading to gain-of-function of this protein (Oyanguren-Desez et al., 2011). A polymorphism in P2X7 receptor T-allele, resulting in an Ala-76 to Val transition (A76V), induced an increase in Ca^2+^ permeability, ethidium bromide uptake, and electrophysiological responses. Also, P2X7 receptor A-allele substitution of His-155 to Tyr-382, increased Ca^2+^ influx (Oyanguren-Desez et al., 2011). Similar findings in P2X7 receptor gain-of-function due to His-155 to Tyr substitution (H155T) was previously described for leukemic lymphocytes (Cabrini et al., 2005) and suggested to be essential for ATP-dependent P2X7 receptor activation, since the residue 155 is important for P2X7 receptor protein folding (Bradley et al., 2011). On the other hand, a genetic study demonstrated that the presence of P2X7 receptor loss-of-function due to an Arg-307 to Gln (N307Q) polymorphism provided a two-fold protective effect against MS outcome (Gu et al., 2015). Thus, human P2X7 receptor variants are associated with a reduced or increased risk of MS development (Oyanguren-Desez et al., 2011; Gu et al., 2015).

*In vivo* studies with the EAE mouse model demonstrated that absence of P2X7 receptors resulted in a severe disease phenotype. In addition, microglia and invading brain macrophages were positive for P2X7 receptor immunostaining (Witting et al., 2006). P2X7 receptor knockout mice (P2X7^−/−^), where EAE was experimentally induced, showed lower number of apoptotic lymphocytes in the CNS and increased expression of interferon γ in the spinal cord, with no alterations in TNF-α and IL-2 protein levels (Chen and Brosnan, 2006). Furthermore, P2X7 receptor^−/−^ EAE mice have lower production of endocannabinoids and reduced axonal damage in comparison to wild type animals (Witting et al., 2006). The administration of a P2X7 receptor antagonist during the chronic phase of EAE in mice attenuated symptoms and tissue damage, including remyelination, by improving axonal conductivity and neurological latency (Matute et al., 2007). These results suggest that the P2X7 receptor plays a detrimental role in the development and chronic phase of MS.

A study by Sharp et al. (2008) demonstrated that the absence of P2X7 receptor results in lower frequency of EAE development, including reduced astrocyte activation with no changes in microglia, antigen responsive T-cell population, or cytokine production by splenic-T cells. These results differ from previous available data on P2X7 receptor ^−/−^ and EAE mice. In the former two studies, deletion of exon 5 in P2X7 receptor was used to derive the knockout mice (Chen and Brosnan, 2006; Witting et al., 2006), while in Sharp et al. (2008) exon 1 was deleted, resulting in macrophages inability to produce IL-1β. Based on these data, regulation of P2X7 receptor activation status can provide beneficial advantages for MS.

Activation of P2X7 receptors in astrocytes induces the release of purines (Ballerini et al., 1996) and limits glutamate removal from the extracellular compartment (Lo et al., 2008), eventually culminating in neuronal/oligodendrocyte excitotoxicity (Pitt et al., 2000; Matute, 2011). Upon stimulation with IL-1β, astrocytes showed P2X7 receptor expression upregulation, indicating that the P2X7 receptor expression depends on the presence of pro-inflammatory cytokines (Narcisse et al., 2005). Furthermore, a hyperactivation of P2X7 receptors in oligodendrocytes causes excitotoxicity by cytosolic Ca^2+^ overload and consequent tissue damage (Matute et al., 2007).

Yiangou et al. (2006) proposed a mechanism for the involvement of P2X7 receptor in MS: increased extracellular ATP levels caused by cell death activate P2X7 receptors in microglia and macrophages, consequently stimulate IL-1β production and release. IL-1β will induce COX2, an enzyme known to be detrimental during inflammation (Minghetti, 2004). This induction will intensify cell death and production of pro-inflammatory cytokines. In addition, in the EAE rat model, protein levels of P2X7 receptor were analyzed at symptomatic manifestation and after recovery (Grygorowicz et al., 2011). During symptom onset, P2X7 receptor was found to be overproduced in synaptosomes and in glial cells homogenates. The elevated protein level of P2X7 receptor was stable at the recovery phase mainly in the glial fraction, suggesting sustained astrogliosis. The use of P2X7 receptor antagonists, such as periodate-oxidized ATP and Brilliant Blue G (BBG), for the treatment of the neurodegenerative phase of MS has been patented (EP1655032 B1), providing novel tools for clinical and research purposes.

#### P2Y receptors

During inflammatory responses, P2Y receptors are up regulated in microglia to promote phagocytosis and migration, preventing oxidative stress followed by apoptosis and controlling the expression of pro-inflammatory cytokines (Förster and Reiser, 2015). In MS, the P2Y12 receptor was found in oligodendrocytes from *post-mortem* brain samples and its expression was decreased in areas corresponding to demyelination in gray and subcortical white matter (Amadio et al., 2010). Immunohistochemistry studies revealed expression of P2Y12, P2Y11, and P2Y14 receptors in the frontal cortex of MS patients (Amadio et al., 2010). However, the functions of P2Y11 and P2Y14 receptors are not known. Therefore, we will further focus on the P2Y12 receptor.

Microglia, macrophages and neuronal cells did not show any expression of P2Y12 receptors, while receptor-positive staining was found to be co-localized with myelin-binding proteins and astrocytes. In the white matter of MS patients, microglia expressing the major histocompatibility complex class II, revealed immunostaining for P2Y12 receptors, indicating that microglia possibly phagocytized myelin-bearing P2Y12 receptors (Amadio et al., 2010). Presence of P2Y12 receptors in astrocytes and oligodendrocytes suggests that signaling of this receptor is involved in remyelination.

To determine the effect of P2Y12 receptor in MS, knockout models of this receptor resulted in an enhanced EAE phenotype in mice (Zhang et al., 2017). The EAE pathology was characterized by an increase in IL-17A cytokine levels in serum, higher number of T-helper cell subset (Th17) in spleen and CNS, as well as the presence of granulocyte-macrophage colony-stimulating factor (Zhang et al., 2017). Bone marrow-derived dendritic cells from P2Y12^−/−^ mice challenged to model EAE have increased release of IL-23, which is an essential factor to promote differentiation of CD4+ T cells toward the Th17 cell subtype (Zhang et al., 2017). The authors concluded that P2Y12 receptors are important for balancing Th-cell populations, and receptor function dysregulation leads to altered cytokine profiles, contributing to EAE.

#### Adenosine receptors

Analysis of peripheral blood mononuclear cells (PBMC) from MS patients showed that A_1_ receptor protein level was significantly reduced (Mayne et al., 1999; Johnston et al., 2001), but gene expression was unaltered (Mayne et al., 1999). In healthy controls, activation of A_1_ receptors in PBMCs resulted in inhibition of TNF-α while in MS patients IL-6 was inhibited and had no effect on TNF-α protein level (Mayne et al., 1999). It has been previously demonstrated that constant presence of high TNF-α levels can induce demyelination in a similar way as observed in MS patients, indicating that dysregulation of TNF-α by A_1_ receptors can be an initiating factor for MS pathology (Probert et al., 1995).

Histological analyses of *post-mortem* brain tissues showed lower expression of A_1_ receptor in the glial population, specifically the A_1_-β receptor spliced variant (Johnston et al., 2001). Induction of the EAE model in ${\text{A}}_{1}^{-/-}$ mice caused a severe progressive-relapsing form of MS with myelin and axonal loss (Tsutsui et al., 2004). Macrophages produced IL-1β and metalloproteinase 12, as well as soluble factors that damaged oligodendrocytes. Analysis of spinal cord of ${\text{A}}_{1}^{-/-}$ EAE mice showed increased release of pro-inflammatory cytokines. In contrast, ${\text{A}}_{1}^{+/+}$ EAE mice had diminished A_1_ receptor expression in microglia, corresponding to inflammation. Chronic caffeine administration upregulated A_1_ receptor expression in microglia, and when treated concomitantly with A_1_ receptor agonist alleviated EAE pathology in the ${\text{A}}_{1}^{+/+}$ EAE mice model (Tsutsui et al., 2004). Furthermore, coffee consumption and MS risk were recently investigated. In individuals, who reported high coffee consumption and in animal models of MS, caffeine decreased the risk of developing neuroinflammation and had neuroprotective and anti-inflammatory properties (Hedström et al., 2016; Olsson et al., 2017).

Current therapeutic recommendations in MS include interferon-β and *glatiramer* (Wiendl et al., 2008). Worthwhile of mentioning, interferon-β treatment increases expression of CD73, responsible for the conversion of AMP into adenosine, in endothelial cells (Airas et al., 2007). Similarly, in an induced-demyelinated rat model, interferon-β treatment also enhanced CD73 activity in synaptosomes from cerebral cortex (Spanevello et al., 2006). CD73 activity is also required for lymphocyte infiltration into the CNS during EAE development (Mills et al., 2008). Thus, interference with levels of purines could be an additional factor, by which interferon-β benefits MS patients.

While the A_1_ receptor subtype is an unequivocal negative modulator of MS and EAE (Tsutsui et al., 2004), the A_2A_ receptor subtype presents a complex role in this disease. Interestingly, A_2A_ receptor expression is increased in the brain of patients with secondary progressive MS, evidenced by positron emission tomography (PET) imaging of radioligand binding to the A_2A_ receptor (Rissanen et al., 2013). This receptor is both highly expressed by lymphocytes and the main mediator of anti-inflammatory effects of adenosine (Blackburn et al., 2009). In the EAE model, A_2A_ receptor-selective antagonist SCH58261 treatment protected mice from EAE induction and CNS lymphocyte infiltration (Mills et al., 2008, 2012). On the other hand, A_2A_ receptor-deficient (${\text{A}}_{2\text{A}}^{-/-}$) mice developed a more severe paralysis after EAE induction, characterized by increased numbers of lymphocytes and activated macrophages/microglia in the CNS (Mills et al., 2012), severe demyelinated phenotype, axonal injury in spinal cord and cerebral cortex and pro-inflammatory cytokine profile in the CNS, blood, and spleen (Yao et al., 2012). Mechanisms of these opposite effects following genetic (knockout animal) or pharmacological (antagonists) blockade of A_2A_ receptors were revealed by assays with bone marrow chimeric mice (subjected to radiation and replacement of immune cells by bone marrow from donor animals) (Mills et al., 2012). This model also reveals the contribution of A_2A_ receptor signaling in immune and non-immune cells during EAE. In fact, ${\text{A}}_{2\text{A}}^{-/-}$ donor hematopoietic cells induced severe EAE, whereas the absence of A_2A_ receptor in non-immune cells protected mice from disease development. Taken together, these data demonstrate that expression of A_2A_ receptors in lymphocytes is crucial for limiting the severity of inflammation, while the A_2A_ receptor on nonimmune cells is necessary for disease development. Moreover, without A_2A_ receptor expression by blood brain barrier cells (and other non-immune cells), immune cells fail to infiltrate the CNS, protecting mice from disease development (Mills et al., 2012), similarly to the effects of pharmacological blockade of A_2A_ receptors (Mills et al., 2008, 2012).

A_3_ receptor signaling is associated with degranulation of mast cells. Activation of A_3_ receptor inhibits adenylate cyclase, stimulates phospholipase C and B, and induces calcium release from intracellular stores. It has been suggested that A_3_ receptors may also inhibit binding of neutrophils to endothelial cells. A_3_ receptor is expressed in whole brain (Safarzadeh et al., 2016). Though it is still unclear whether the A_3_ receptor is involved in MS, this receptor has been demonstrated to mediate the inhibition of TNF-α production by adenosine (Lee et al., 2006; Levy et al., 2006). Therefore, this receptor may play important roles in the pathophysiology of MS and, such as for the A_2A_ receptor, A_3_ receptor inhibition may be a potential therapeutic approach.

A_2B_ receptor signaling also modulates the pathogenesis of EAE phenotype. This receptor is upregulated in peripheral leukocytes of MS patients and in the mouse model. Activity inhibition of A_2B_ receptor with the selective antagonist CVT-6883 or its genetic deletion attenuated adenosine-mediated IL-6 production, infiltration of peripheral leukocytes and clinical symptoms in the EAE model (Wei et al., 2013). The presented studies suggest that adenosinergic activation of A_1_ receptor regulating inflammatory cytokine TNF-α and IL-6 production is altered in MS, probably due to alterations at transcriptional levels of A_1_ receptor and/or to adenosine availability (Mayne et al., 1999; Johnston et al., 2001).

#### Conclusion

Since current therapeutic recommendations for MS have partial efficacy on clinical outcomes and disease progression, the search for new therapeutic tools is necessary. In this context, *post-mortem* analysis of brain tissue from both MS patients and EAE mouse/rat models elicited potential therapeutic targets through: (1) blockade of P2X7 receptor and stimulation of A_1_ receptor, inhibiting inflammation; (2) P2Y12 receptor stimulation, favoring remyelination; and (3) blockade of A_2B_ receptors and CD73, inhibiting the infiltration of leukocytes into the CNS.

## Parkinson's disease

Parkinson's Disease (PD) is the second most common neurodegenerative disease. Its incidence increases with age reaching over 4% of the population over 80 years old (de Lau and Breteler, 2006). PD is considered a motor disease as a reflex of its clinical symptoms, as resting tremors of extremities, muscular rigidity, postural imbalance, and bradykinesia (Braak et al., 2013). The pathology of PD is characterized by progressive loss of dopaminergic neurons in the *substantia nigra pars compacta* (SNc) and their projections to the striatum, structures associated to voluntary motor movements' control. Besides dopaminergic neuronal degeneration, the presence of protein aggregates (known as Lewy bodies) due to misfolding of α-synuclein occurs in the SNc, *locus ceruleus*, amygdala, and the CA2 area of the hippocampus (Jellinger, 2011). The mechanisms underlying these events have yet to be clarified, although a genetic predisposition associated with insults as traumatic brain injury and ischemia seems to induce α-synuclein aggregation (Shahaduzzaman et al., 2013; Kim and Vemuganti, 2017). The majority of genes linked to familial PD development, such as α-synuclein and leucine-rich repeat kinase-2 (LRRK2) apparently follow a non-Mendelian genetic inheritance pattern. Even so, people who have first-degree relatives affected by sporadic PD have increased chances of developing PD (Elbaz et al., 1999). Mitochondrial dysfunction and purinergic receptor signaling are also involved in the mechanism of the disorder (Takenouchi et al., 2010; Hoang, 2014).

Currently, dopamine agonists as L-3,4-dihydroxyphenylalanine (L-DOPA) are the most common agents used in therapy. L-DOPA is a precursor of catecholamines such as dopamine and is able to cross the blood-brain barrier. However, long term use of L-DOPA loses efficacy and dose adjustments are needed, triggering side effects such as dyskinesias in 50% of patients after 5 years of continuous treatments (Lang, 2009; Olanow et al., 2009). Present studies on molecular aspects of PD, together with the development of new drugs and tests for improving diagnosis accuracy, will bring new therapeutics perspectives for the disease.

### Purinergic involvement in PD

#### P2X receptors

Although immunohistochemistry analysis did not reveal any difference between intact and lesioned striatum and SNc (Amadio et al., 2007) for P2X7 receptors, antagonism of this receptor has been shown to prevent or reverse hemiparkinsonian behavior in animals lesioned with 6-hydroxydopamine (6-OHDA), a neurotoxin that mimics PD's pathology. Acute SNc injections of the P2X7 receptor antagonist A-438059, 60 min before and 60 min after rat 6-OHDA lesion, prevented dopamine striatal deficit in comparison to the intact hemisphere, with the P2X7 receptor localized in glial cells (Marcellino et al., 2010). BBG administered in a dose of 45 mg/kg daily after 6-OHDA lesion prevented hemiparkinsonian behavior, short-term memory impairment and dopamine deficit in the striatum and SNc (Carmo et al., 2014). While these studies showed only a preventive effect of P2X7 receptor antagonism, BBG at a dose of 50 mg/kg reversed 6-OHDA lesion in striatum and SNc. In this work, BBG treatment started 1 week after 6-OHDA injection, a period of time sufficient for the lesion to settle, thus proving the reversal effect (Ferrazoli et al., 2017).

Neuronal death seems to aggravate protein aggregation observed in PD. Intense ATP release and consequent purinergic receptors activation were considered to be a key trigger. In fact, P2X1 receptor antagonism or genetic deletion reduced α-synuclein aggregation induced by ATP released by dying cells *in vitro* (Gan et al., 2015). Moreover, P2X1 receptor activation induced lysosomal dysfunction that seems to be involved in α-synuclein aggregation, since it delayed protein turnover and led to its accumulation (Gan et al., 2015). Although P2X7 receptor blockade did not result in reduction of α-synuclein aggregation in this study, ATP release triggered by α-synuclein *in vitro* activated the P2X7 receptor and mobilized the release of intracellular Ca^2+^, showing that P2X7 receptor activation is a consequence of α-synuclein aggregation (Wilkaniec et al., 2017). Additionally, another study showed that microglial cells challenged with α-synuclein presented increased ROS production through P2X7 receptor activation, which was prevented in the presence of a receptor antagonist (Jiang et al., 2015). Thus, it seems that P2X1 receptor activation contributes to α-synuclein aggregation, which in turn modulates P2X7 receptor activity, ROS production and, finally, ATP release.

Taking into account that few studies directly link purinergic receptors with genetic predisposition to PD, the P2X7R 1513A>C polymorphism that facilitates pore formation by P2X7 receptor activation and leads to cell death (Gu et al., 2001) was shown to be a risk factor in sporadic PD in a Han Chinese population (Liu et al., 2013)

#### P2Y receptors

There is little data directly connecting P2Y receptors to PD. Recent studies are drawing attention to the role of P2Y6 receptors in PD development and progression. An *in vitro* study showed that P2Y6 receptor gene expression is increased in SH-SY5Y cells—a human neuroblastoma lineage that when differentiated presents markers for dopaminergic neurons—when challenged with neurotoxin 1-methyl-4-phenylpyridinium (MPP^+^) (Qian et al., 2017). Thus, its antagonism or deletion decreased MPP^+^ effects in cell death through reduced ROS production (Yang et al., 2017). In the CNS, UDP released by damaged cells induces expression of cytokines CCL2 and CCL3 in microglia and phagocytic activity through activation of P2Y6 receptors, indicating that this receptor subtype may be involved in inflammatory response in neurodegenerative diseases (Kim et al., 2011).

Recently, P2Y6 receptor levels were found to be increased in PBMC of PD patients younger than 80 years. To elucidate the involvement of P2Y6 receptor in these patients, the authors used an *in vitro* model of microglia challenged with lipopolysaccharide (LPS) and found increased P2Y6 receptor expression, supporting the hypothesized neuroinflammatory effect of microglia (Yang et al., 2017). Taking into account that P2Y6 receptor selective inhibition by MRS2578 is able to prevent microglial phagoptosis in a mixed neuronal/glial culture in inflammatory conditions (Neher et al., 2014), P2Y6 receptor antagonism seems to be a promising tool to attenuate neuronal death in PD by preventing lesion worsening due to phagocytosis of viable neurons.

#### Adenosine receptors

It is known that A_2A_ receptors are enriched in dopaminergic brain areas and that their activity modulation affects dopamine receptors (Burnstock et al., 2011). In fact, A_2A_ receptors form heterodimers with dopaminergic D2 and A_1_ receptors in glutamatergic synapses, modulating the balance between excitatory and inhibitory impulses that may aggravate PD symptomatology (reviewed by Schiffmann et al., 2007). In animals, a range of A_2A_ receptor antagonists have being shown to potentiate therapeutic effect of low doses of L-DOPA in MPP^+^ lesioned monkeys and marmosets and in 6-OHDA lesioned rodents (Kanda et al., 2000; Fuzzati-Armentero et al., 2015) In fact *istradefylline*, a A_2A_ receptor antagonist, was recently approved in Japan to be used concomitantly with L-DOPA treatment, once the compound enhances antiparkinsonian effect of L-DOPA and allow the usage of lower doses of L-DOPA with less long-term side effects (Zhu et al., 2014).

A_2A_ receptors are supposedly involved in synucleinopathy process. A_2A_ receptor-knock out mice presented resistance in preventing dopaminergic deficits upon α-synuclein-induced insults (Kachroo and Schwarzschild, 2012). Attempting to clarify involved mechanisms, Ferreira et al. found that A_2A_ receptor antagonism decreased α-synuclein aggregation, prevented neuronal death induced by extracellular α-synuclein and restrained hyperactivation of NMDA-glutamate receptors (Ferreira et al., 2015). A_2A_ receptor protein expression levels are increased upon hippocampal injections of α-synuclein in mice and closely co-localized with aggregates, suggesting a pathogenic role of this receptor in synucleinopathy (Hu et al., 2016).

Moreover, A_2A_ receptor antagonism may facilitate microglial response to injury. Microglial delayed containment of debris resulted from cell death can be associated with expansion of the lesion (Gyoneva et al., 2014). Further, both caffeine and selective A_2A_ receptor antagonist KW60002 prevented rat striatal dopaminergic deficit and hydroxyl radical release in LPS-induced inflammation (Gołembiowska et al., 2013). These data suggest inflammatory modulation by A_2A_ receptor antagonism in PD models.

Two polymorphisms of A_2A_ receptor (rs71651683 or rs5996696) were inversely associated with genetic PD risk, wherein caffeine intake intensified the inverse association. Moreover, two polymorphisms in CYP1A2a (rs762551 or rs2470890), an enzyme responsible for caffeine metabolism, in homozygous caffeine consumers showed a prominent reduction in the risk of developing PD (Popat et al., 2011).

Caffeine intake interferes with other genetic risk factors for PD. Subjects with LRRK2 risk variant R1628P showed 15 times increased risk of developing PD than not caffeine consumers (Kumar et al., 2015). GRIN2A rs4998386-T allele encodes a subtype of NMDA receptor, whose activity is enhanced by A_2A_ receptor activation and leads to glutamatergic excitotoxicity. A polymorphism in the GRIN2A rs4998386-T is considered protective for PD development *per se*, but in association with caffeine consumption, it can beneficially impact PD risk in a greater magnitude (Hamza et al., 2011; Yamada-Fowler et al., 2014). However, creatine consumption that increases ATP storage accelerated PD progression in GRIN2A caffeine consumers, possibly due to ATP conversion to adenosine and later A_2A_ receptor activation (Simon et al., 2017).

#### Conclusion

Taken together, evidence indicates that modulation of purinergic receptor expression and activity could be useful in PD treatment in several ways: (1) reducing microglia activation by damaged cells and α-synuclein aggregation through P2X7 and P2Y6 receptors antagonism; (2) preventing α-synuclein aggregation through P2X1 and A_2A_ receptors antagonism; (3) modulating inflammatory scenario through A_2A_ receptors antagonism; or (4) preventing dyskinesia induced by L-DOPA long-term use through combined treatment with A_2A_ receptor antagonists.

## Other neurological conditions with motor dysfunctions

### Huntington's disease

HD is an inherited neurological disorder caused by a mutation in IT15 gene that encodes huntingtin protein (Htt) predominantly found in neurons. This mutation results in abnormal (CAG)n repeats localized in 5′ coding sequence. HD is characterized by neurodegeneration of neuronal cells located in striatum and cerebral cortex, ultimately causing neuronal dysfunction and striatal death (Vonsattel and DiFiglia, 1998; Ross and Tabrizi, 2011).

#### Purinergic involvement in HD

##### Adenosine receptor

Adenosinergic pathway plays an essential role in HD etiology and progression, especially through the A_2A_ receptor, as observed in patients and animal models (Popoli et al., 2007). The A_2A_ receptor is highly expressed in striatum (Schiffmann et al., 1991; Fink et al., 1992) especially in GABAergic/enkephalinergic neurons (Taherzadeh-Fard et al., 2010) and in post-synaptic striatopallidal GABAergic neurons (Martinez-Mir et al., 1991; Hettinger et al., 2001), antagonizing dopamine D2 receptors (Schiffmann et al., 2007), while presynaptic A_2A_ receptor activity promotes glutamate release (Shen et al., 2013). Further, presynaptic A_2A_ receptors in glutamatergic terminals impinging into medium spiny neurons play an essential role in the initial maladaptive plasticity in animal models of HD (Li et al., 2015), suggesting its involvement in the degeneration of striatal neurons. Reduction of A_2A_ receptor expression is based on the overexpression of mutant Htt protein showing expanded poly (Q), which affects CREB binding to its promoter region in the A_2A_ receptor gene. Under stimulation, A_2A_ receptor is able to promote its own gene expression via activation of PKC/CREB signaling as well as reduce Htt aggregations (Chiang et al., 2005). Striatal cells expressing mutant Htt showed increased A_2A_ receptor density and cAMP activity due to A_2A_ receptor activation (Varani et al., 2001). As expected, transgenic HD mice showed reduced A_2A_ receptor expression (Cha et al., 1999; Glass et al., 2000; Luthi-Carter et al., 2000), while exhibiting transient increases in A_2A_ receptor density and A_2A_ receptor-dependent activation of cAMP signaling at the earlier pre-symptomatic stage (Tarditi et al., 2006).

It has been proposed that modulation of A_2A_ receptor activity either by agonists or antagonists may prove to be beneficial for HD treatment. However, available data indicate that the beneficial effect observed after stimulation or inhibition of A_2A_ receptor activity depends on the disease stage. At earlier stages of HD, the use of SCH58261 (an A_2A_ receptor antagonist) in quinolinic acid (QA)-induced HD rats and R6/2 transgenic mice reduced striatal BDNF expression, precluding BDNF control of NMDA toxicity (Potenza et al., 2007; Tebano et al., 2010). In later stages, no effect on BDNF expression was observed (Martire et al., 2010; Tebano et al., 2010). QA-induced rats reproduced neurochemical changes of NMDA receptor from HD, e.g., increased glutamate outflow, reduced adenosine levels and degeneration of A_2A_ and dopamine receptors (Beal et al., 1991; Ishiwata et al., 2002; Gianfriddo et al., 2003). Treatment with SCH58261 2-3 weeks after QA injection increased striatal glutamate release, acting as damaging factor (Gianfriddo et al., 2003).

Preventive treatment with SCH58261 before QA induction in rats minimizes the effect of QA on motor activity, striatal gliosis, electroencephalographic (EEG) changes, and glutamate levels (Popoli et al., 2002). However, cyclooxygenase-2 (COX-2) is inhibited in microglia but increased in cortical neurons, probably as a consequence of NMDA receptors activation, leading to neurotoxicity (Minghetti, 2004). Pretreated QA-induced rats also showed less rearing behavior and no changes in baseline motor activity after 2 weeks of induction; 6 months later, rats showed reduced anxiety but no changes in learning task when compared to QA-induced rats not pre-treated with SCH58261 (Scattoni et al., 2007). These findings suggest that SCH58261 acts on damaged striatum and not on damaged hippocampus, and that different populations of striatal neurons are responsive to SCH58261 (Scattoni et al., 2007). As reviewed by Cunha (Cunha, 2016), the blockage of A_2A_ receptor improves memory and motor functions indicating hippocampal activity, contradicting the findings of Scattoni et al.

On the other hand, in primary striatal cultures treated with QA, an increase in intracellular calcium concentration was observed which enhanced in presence of SCH58261, but reduced in presence of A_2A_ receptor agonist CGS21680 (Popoli et al., 2002). Another A_2A_ receptor antagonist, 3,7-dimethyl-1-propargylxanthine (DMPX), completely blocked encephalographic changes in prefrontal cortex in QA-induced rats (Reggio et al., 1999). The beneficial effect can be due to dopamine receptor activation that provides neuroprotection as a result of abolishment of A_2A_ receptor function, since D2 dopaminergic receptors are downregulated by A_2A_ receptors in D2/A_2A_ receptor heteromers (Reggio et al., 1999).

In a transgenic rat model of HD showing 51 repeated CAG sequences, the presence of post-synaptic A_2A_ receptor antagonist KW-6002, a known stimulant of locomotion, didn't alter the locomotion pattern between 3 and 6 months old. This indicated that the animals become indifferent to A_2A_ receptor modulation during that period (Orr,ú et al., 2011). Furthermore, the presynaptic A_2A_ receptor antagonist SCH-442416 did not reduce electromyography responses (Orr,ú et al., 2011).

The function of A_2A_ receptors has been studied in HD transgenic mouse models (R6/1 with later symptoms and R6/2 with earlier symptoms), which contain the first exon of human Htt gene and 115-150 CAG repeats (Li et al., 2005). During R6/2 mouse development, A_2A_ receptor protein density and A_2A_ receptor-dependent production of cAMP slightly increased at post-natal days 7–14, before the onset of motor symptoms (Tarditi et al., 2006). On the 21st day, changes are normalized to control (Tarditi et al., 2006). A_2A_ receptor expression, but not protein density, starts decreasing, indicating that protein turnover is altered in HD (Cha et al., 1999; Tarditi et al., 2006). Reduction of A_2A_ receptor coding mRNA can be explained by regulation of A_2A_ receptor gene methylation patterns, once R6/1 mice has less hydroxymethylcytosine and higher methylcytosine levels in 5′-UTR regions of the A_2A_ receptor gene (Villar-Menéndez et al., 2013).

Since turnover of A_2A_ receptor protein is altered in HD, inhibition of this receptor function is an advisable therapeutic approach. Starting at 5 weeks, the use of the A_2A_ receptor antagonist SCH58261 in R6/2 mice ameliorated NMDA-induced toxicity and emotional/anxiety response (Domenici et al., 2007). After week 8, administration of SCH58261 leads to NMDA receptors remodeling (NR_1_ and NR_2A_ receptor /NR_2B_ ratio) in striatum (Martire et al., 2010). R6/2 mice at age of 10–11 weeks old showed increased adenosine levels correlated with the presence of p38 MAPK in striatal neurons, resulting in striatal damage (Gianfriddo et al., 2004). The usage of SCH58261 greatly reduced striatal adenosine levels and glutamate outflow, suggesting that SCH58261 was acting on A_2A_ receptors located in corticostriatal glutamatergic terminals (Gianfriddo et al., 2004). When treated with SCH58261, rearing and grooming behaviors were reduced in R6/2 mice, but increased in wild type mice, suggesting that A_2A_ receptor antagonism effects on behavior depended on the presence of mutant Htt (Domenici et al., 2007). However, there are contradictory findings regarding the effect of SCH58261. While this compound has shown beneficial effect by reducing NMDA toxicity in striatum *in vivo*, it did not prevent NMDA toxicity from *in vitro* culture of corticostriatal slices obtained from R6/2 mice (Martire et al., 2010).

In order to determine whether the A_2A_ receptor is involved in HD etiology, A_2A_ receptor knockout mice were induced with mitochondrial toxin 3-nitropropionic acid (3-NPA) which blocks succinate dehydrogenase, inducing HD phenotype. Only 1 out of 8 showed striatal lesion after 3-NPA induction, indicating that the absence of A_2A_ receptor has protective effect against HD development (Fink et al., 2004). To confirm this finding, wild type mice were pre-treated with the A_2A_ receptor antagonist 8-(3-chlorostypyl)-caffeine. The animals did not show any striatal lesions after 3-NPA treatment (Fink et al., 2004). On the other hand, ablation of A_2A_ receptors in HD N171-82Q transgenic mouse model completely aggravated motor performance and survival, reducing the expression of striatal encephalin (Mievis et al., 2011). This observation suggests that early and chronic blockade of A_2A_ receptor is not favorable for HD development (Mievis et al., 2011), but memory improvement was observed in R6/2 mice with complete genetic A_2A_ receptor ablation (Li et al., 2015).

In symptomatic R6/2 mice, activation of A_2A_ receptors by CGS21680 delayed the deterioration of motor conditions, prevented reduction in brain weight, diminished the levels of choline, normalized glucose levels, and altered NMDA receptor subunit composition and basal synaptic transmission, without changing its expression (Chou et al., 2005; Martire et al., 2007; Potenza et al., 2007; Ferrante et al., 2010; Tebano et al., 2010). Cultivation of corticostriatal slices from R6/2 mice in presence of CGS21680 also showed reduced NMDA toxicity, suggesting a crosstalk between A_2A_ receptor and BDNF (Tebano et al., 2010). Treatment of striatum slices from R6/2 with CGS21680 resulted in an increase in extracellular field potential, while the opposite effect was observed in wild type slices, where the use of an A_2A_ receptor agonist potentiated toxicity via NMDA receptor activation (Martire et al., 2007).

Single nucleotide polymorphisms (SNPs) in the ADORA_2A_ gene have been identified in HD patients. A C>T genotype (1876 C/T; rs5751876) SNP results in a silent mutation with unknown function and influences the age of onset of HD, while the T/T genotype increases the age of onset of HD by 3.8 years when compared to the C/C genotype (Dhaenens et al., 2009). A SNP in intron 1 (rs2298383) is linked to early onset of HD (Taherzadeh-Fard et al., 2010). Analysis of HD patient peripheral blood cells led to increased aberrant A_2A_ receptor signaling, which correlates with the age of the patient, numbers of expanded CAG repeats and number of A_2A_ receptor ligand-binding sites (Maglione et al., 2005a,b). The linear correlation is more evident in patients suffering from chorea—an early disruption of the striatum in HD. Neutrophils from HD patients have higher A_2A_ receptor dysfunction in homozygous vs. heterozygous HD patients while no changes in A_1_ or A_3_ receptors are observed in peripheral blood cells (Varani et al., 2003). A_2A_-cannabinoid CB1 receptor heterodimers exert crucial function by controlling neuronal excitability (Moreno et al., 2017), while activation of striatal A_2A_ receptors may inhibit CB1 function independent from heterodimer formation (Ferreira et al., 2015). Patients harboring high-grade HD do not possess A_2A_-CB1 receptor heterodimers in the caudate-putamen region due to the lack of CB1 receptors (Moreno et al., 2017). Recent evidence suggests that consuming more than 190 mg/day of caffeine may accelerate HD onset (Simonin et al., 2013), contradicting findings in animal models that point toward beneficial effects of A_2A_ receptor antagonism in HD.

HD is also characterized by oxidative stress resulting from mitochondrial dysfunction, leading to GABAergic neuronal loss and proneness to DNA damage (Chiu et al., 2015). GABAergic neurons derived from HD-iPSC showed an increase in DNA damage and oxidative stress, which can be dramatically reduced by A_2A_ receptor activation (Chiu et al., 2015). Stimulation of A_2A_ receptors minimizes oxidative stress-induced apoptosis by activation of the cAMP/PKA signaling pathway (Chiu et al., 2015), which is essential for reversing the effect of reduced A_2A_ receptor activity via CREB transcription factor activation (Chiang et al., 2005). However, findings *in vivo* contradict the beneficial effect of A_2A_ receptor agonism on PKA signaling. In R6/1 mice, dopamine D1 and A_2A_ receptors are hyperactive showing greater cAMP/PKA signaling (Tyebji et al., 2015). Chronic administration of antagonists of dopamine D1 and A_2A_ receptors normalized PKA levels and improved cognitive dysfunction and synaptic plasticity. Pre-treatment of rats and mice with either 8-cyclopentyl-1,3-dipropylxanthine (CPX; A_1_ receptor antagonist) or DMPX prior application of manolate (an inhibitor of mitochondria acting in striatum) showed that DMPX prevented GABAergic cell loss while CPX promotes cell death (Alfinito et al., 2003). The A_1_ receptor agonist R-PIA prevented seizures but not neurodegeneration in the 3-nitropropionic acid (3-NPA) model of neurotoxicity (Zuchora and Urbañska, 2001), while the A_1_ receptor agonist adenosine amine congener (ADAC) protects against excitotoxicity, delays degeneration and improves motor functions in the same model (Blum et al., 2002). In view of that, the effect of A_1_ receptors depended on the respective used antagonist.

##### P2 receptors

The role of P2X signaling in HD has not yet been studied in detail. Evidence exists that signaling via ATP induced cell death in HD models while blockade of ATP production reduces cell loss (Varma et al., 2007). At the present, the only evidence available is the role of P2X7 receptor in HD pathogenesis. In two HD mice model, Tet/HD94 and R6/1, P2X7 receptor expression is increased, as well as P2X7 receptor-induced Ca^2+^ permeability (Diaz-Hernandez et al., 2009). Treatment with the P2X7 receptor antagonist BBG ameliorates motor coordination deficits and body weight loss while inhibiting neuronal loss. *In vitro*, neurons expressing mutant Htt are prone to cell death induction by apoptosis after P2X7 receptor stimulation (Diaz-Hernandez et al., 2009).

##### Conclusion

The available data on the involvement of A_2A_ receptors in HD progression is evident, suggesting that the prevention of its activation could delay disease progression. Taken together, it can be proposed that a combination of A_1_ receptor agonist and A_2A_ receptor antagonist might be a good therapeutic approach for HD. It must be taken in consideration that the effect of A_2A_ receptor antagonism depends on age, doses, and length of treatment. Although antagonism of P2X7 receptor may be promising, the involvement of other P2 receptors remains unclear and needs to be investigated.

### Ataxias

Ataxia, or dysfunction in motor coordination, is a major consequence of cerebellar and spinocerebellar tract dysfunction that can be induced by several factors, including genetic and sporadic forms, commonly related to immune system mechanisms (Mariotti et al., 2005). Spinocerebellar ataxia (SCA), a genetic-related form of progressive ataxia resulted by cerebellar degeneration, is classified according to mode of inheritance and gene/chromosome locus affected (Matilla-Dueñas et al., 2012). The most prevalent and severe forms of SCA are caused by an increase in CAG sequence repeats in genes that encode proteins related to disease development (Paulson et al., 2017). For example, the expansion in polyglutamine affecting ataxin-2 protein can be observed in SCA type 2, while ataxin-3 related expansion occurs in SCA type 3. Other forms of SCA can be characterized by other genetic mutations, such as the type 14, in which mutations in the protein kinase C-γ gene induce cerebellar degeneration (Seki et al., 2005).

#### Purinergic involvement in ataxias

Attempting to identify survival characteristics of some cell in SCA type 2, wild type ataxin-2 positive neurons showed resistance in cell-death induced by axotomy (Viscomi et al., 2005) and, although this lesion up-regulated P2X1 and P2X2 receptors in precerebellar nuclei (Florenzano et al., 2002) and induced P2X1 receptor in ataxin-2 positive neurons, the percentage of cells expressing P2X1 receptor was not altered (Viscomi et al., 2005). Viscomi and co-workers suggested that these purinergic receptors could influence resistance against cell death without being essential for cell survival, since there are several pathways involved in neuronal death. The elucidation of purinergic receptor involvement in SCA type 3 is focused on adenosine receptors. The blockade of A_2A_ receptors through caffeine ingestion reduced damaging morphological changes induced by mutant ataxin-3 injection. Moreover, these damaging effects were abolished in knockout mice for A_2A_ receptors (Gonçalves et al., 2013). Behavioral improvements were also observed in transgenic c57Bl6 mice expressing truncated polyglutamine ataxin-3 with severe ataxia, reinforcing the protective effect of A_2A_ receptor antagonism in the SCA type 3 (Gonçalves et al., 2017). In the SCA type 14 *in vitro* model, stimulation of purinergic receptors with ATP transiently increased translocation of mutant protein kinase C-γ to the plasma membrane and subsequent increased damaging aggregation in the cytoplasm (Seki et al., 2005).

### Restless leg syndrome

Restless leg syndrome (RLS) is a neurological condition characterized by an urge to move legs during rest, following a circadian cycle with worsening during night and even during sleep (named periodic limb movements of sleep). Pathophysiological mechanisms have not been fully elucidated, and conflicting results are reported in the literature. Dopaminergic transmission seems to be involved, since the use of dopaminergic-inducing drugs improved symptoms (Garcia-Borreguero and Cano-Pumarega, 2017). Due to the high affinity of the agonists with best responsiveness to RLS for D3 dopaminergic receptors, it is postulated that the D3 receptor subtype has major responsibility for RLS improvement (Ferré et al., 2018). However, the risk of symptoms worsening after long-term use of these drugs stimulated the search for alternative therapies, based on glutamatergic ligands and reversal of iron deficiency (Ferré et al., 2017). Striatal glutamatergic terminals are found to be hypersensitive in an animal model of RLS with increased glutamate and dopamine release. It is known that, besides increased dopamine release, there is a decreased synaptic D2 receptor density in this animal model (Ferré et al., 2018).

#### Purinergic involvement in RLS

Recently, Ferré's group pointed at a relation between adenosine receptors and brain iron deficiency in RLS. Using an animal model for RLS, in which mice and rats adhered to an iron deficient diet, striatal presynaptic A_2A_ receptor density was upregulated (Gulyani et al., 2009). In the same animal model, A_1_ receptor density was found decreased in animals with mild, moderate and severe deficiency accompanied by dopaminergic D2 receptor downregulation, and increased pre-synaptic A_2A_ receptor density in animals submitted to a more iron deficient diet (Quiroz et al., 2016). Thus, the post-synaptic A_1_ receptor, which can be found as heteromers with D1 dopaminergic receptors and antagonizes their activity, as well as presynaptic A_2A_ receptors forming heteromers with D2 receptors whose activation decreases D2 receptor affinity for agonists, could be targets to improve movement impairment (Ferré et al., 1994; Ferre et al., 1996; Ferré et al., 2007, 2018). Finally, A_1_/A_2A_ receptor heteromers found in the striatal glutamatergic terminals, activated by different adenosine concentrations, decreased glutamate release, a condition found in brain iron deficiency animals (Ciruela et al., 2006; Ferré, 2010).

## Outcomes for hypotheses on P2 purinergic signaling

**Figure 2:**
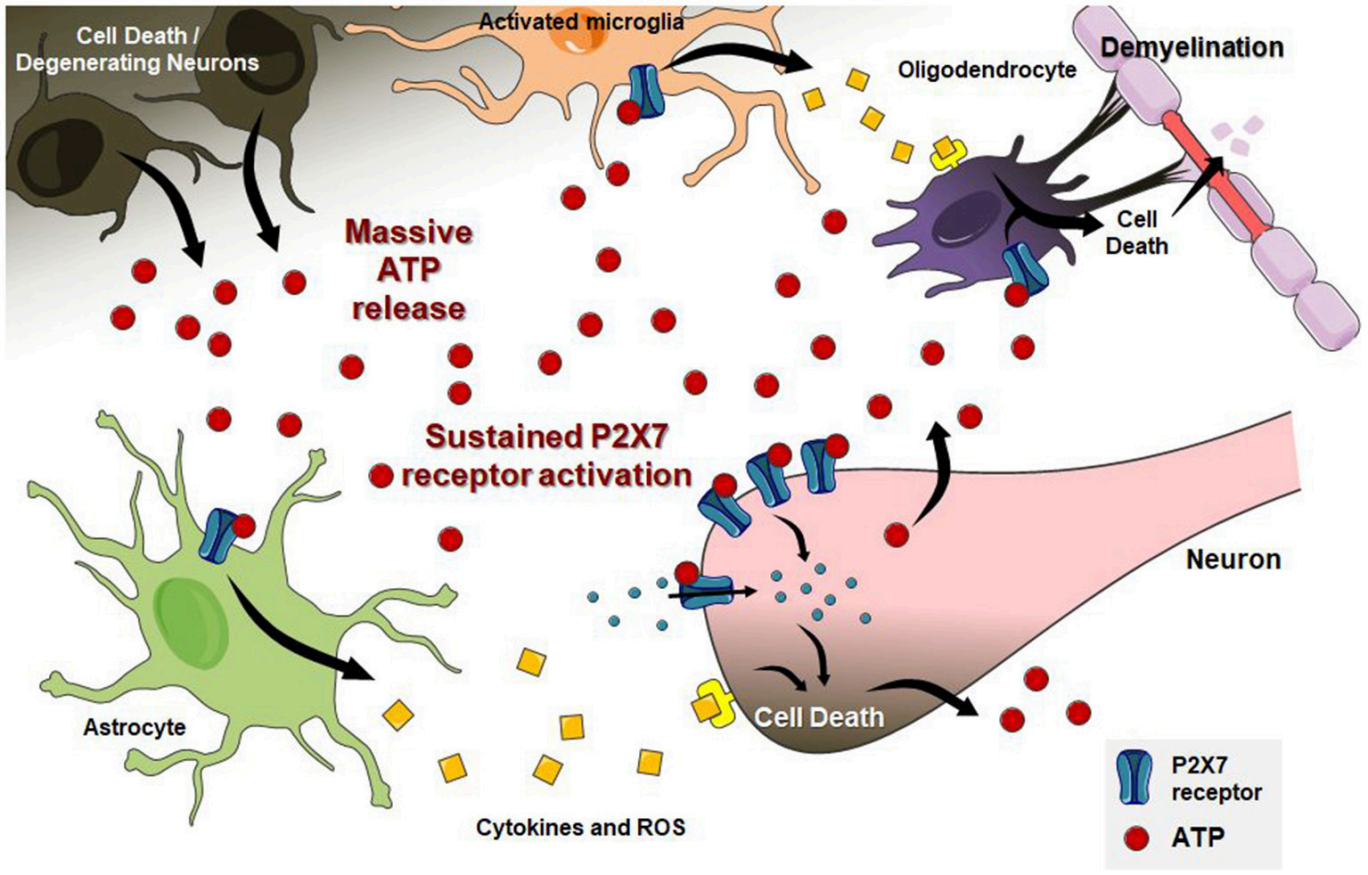
Common mechanism involving P2X7 receptor-mediated cell death in the central nervous system under neurological diseases affecting motor functions. Degenerating neurons release large amounts of ATP, leading to sustained P2X7 receptor activation in: a. Astrocytes, inducing the release of cytokines and reactive oxygen species (ROS); b. Microglia, inducing an activated state and release of cytokines and ROS; c. Oligodendrocytes, inducing cell death and neuron demyelination; d. Neurons (in spite of the controversial discussion on expression of P2X7 receptors in this cell type), inducing pore formation, ion influx and cell death, releasing more ATP into the extracellular space. Moreover, cytokines and ROS released by astrocytes and microglia act on other neural cells, culminating in apoptotic pathway activation.

Although P2X7 receptor expression and levels in neurons is controversial, the involvement of this receptor in neurodegeneration is well-stated (Illes et al., 2017). Of all purinergic receptors, the P2X7 receptor has the lowest affinity for ATP, and only high concentrations of this nucleotide induce channel formation (North, 2002; Khakh and Alan North, 2006). Of the neurological diseases presented here, immune system responses and neural cell death correspond with the release of elevated levels of ATP into the extracellular space. In these scenarios, ATP in excess acts as a toxin that can directly induce oligodendrocyte death by activating P2X7 receptors, resulting in progressive neural damage (Matute et al., 2007; Domercq et al., 2009). Corroborating this idea, it is well-known that the P2X7 receptor triggers pro-inflammatory effects (Lister et al., 2007), and its antagonism can counteract chronic inflammation observed in these diseases (Figure 2). Thus, beneficial effects of P2X7 receptor antagonism are of interest for future therapeutic approaches.

As further investigations are necessary to better understand the real role of purinergic signaling in the diseases here presented, we also propose a novel mechanistic perspective, in which purinergic receptors from glial cells are key initiators of motor dysfunction in PD, MS, ALS and other MND. Although there is no data regarding P2Y1 receptor functions in these pathological conditions, it is known that this receptor plays a crucial role in astrocyte responses accompanied by P2Y12 and adenosine receptor activity modulation (Mamedova et al., 2006).

During an initial inflammatory response, microglial activation induces P2Y12 receptor expression level and/or activity upregulation, stimulating motility toward the injury site and resulting in reduced P2Y1 receptor expression in astrocytes. This downregulation in P2Y1 receptor expression stimulates an increase in reactive astrogliosis and a phenotypical change in order to promote neuroprotection (Haynes et al., 2006; Mamedova et al., 2006; Shinozaki et al., 2017). However, constant inflammatory responses disable microglia of stimulating P2Y12 receptor expression and activity, resulting in a permanent activation of P2Y1 receptors in astrocytes and extended ROS production (Rodrigues et al., 2015). This condition will lead to a downregulation of A_1_ receptor expression, stimulating TNF-α production and release, thereby promoting IL-6 secretion through A_2B_ receptor activation. As a result, TNF-α and IL-6 accumulation damages oligodendrocytes and provoke demyelination. A scheme of this mechanism is proposed in Figure 1.

## Overall conclusion

The purinergic signaling system has risen in the past years as a meaningful research object for understanding and treating several pathologies, as reviewed by Burnstock (2017). Purinergic receptors and enzymes are in the spotlight for new therapeutic interventions as key regulators of neuron-glia communication, as well as modulators of many signaling pathways associated to neuroprotection, neurodegeneration, and neuroregeneration (Burnstock, 2016; Ribeiro et al., 2016). In this review, we highlighted available data linking purinergic signaling pathways to neurological diseases, such as PD, MS, ALS, and other MND, putting together published knowledge with novel hypotheses for overcoming motor dysfunctions. The evidence is summarized in Table 1. A common mechanism supporting P2X7 receptor hyperactivation through high levels of ATP release during disease development is illustrated in Figure 2. Moreover, a novel mechanism based on purinergic modulation of glial cells is proposed in Figure 1. These approaches suggest novel possible research targets to understand the here presented motor dysfunctions and other factors associated to neurological impairment that have not been studied yet. Applied research will need to be conducted for the development of novel pharmacological treatments to improve patients' lifespan and quality.

## Author contributions

ÁO-G: Contribution to idea proposal, organization of sections, writing PD, other MND, RLS, and Ataxia sections, figures, table. YN: Writing MS, HD, and mechanism hypothesis sections. LS-A: Writing ALS and SMA sections. MG: Writing introduction, HD, and conclusion sections. JC-V: Writing ALS section. MP: Writing MS section. HdS: Writing PD section. HU: Conceptualization of the manuscript, supervision of manuscript elaboration, editing and revision, and critical overview.

### Conflict of interest statement

The authors declare that the research was conducted in the absence of any commercial or financial relationships that could be construed as a potential conflict of interest.

## Abbreviations

6-OHDA: 6-hydroxydopamine
ALS: amyotrophic lateral sclerosis
ATP, ADP, AMP: adenosine tri-, di-, monophosphate
BBG: Brilliant Blue G
BDNF: brain-derived neurotrophic factor
BzATP: 2′-3′-O-(benzoyl-benzoyl) ATP
cAMP: cyclic AMP
CNS: central nervous system
COX2: cyclooxigenase 2
EAE: encephalomyelitis
HD: Huntington Disease
Htt: huntingtin protein
IL-1β/2/6/17A/23: Interleukin 1β/2/6/17A/23
iPSC: induced pluripotent stem cells
L-DOPA: L-3,4-dihydroxyphenylalanine
LPS: lipopolysaccharide
MAPK/ERK: mitogen activated protein kinases/extracellular signal-regulated kinases signaling induction pathway
MNs: motor neurons
MND: motor neuron diseases
MPP+: 1-methyl-4-phenylpyridinium
MS: multiple sclerosis
NOX2: nicotinamide adenine dinucleotide phosphate-oxidase 2
PBMC: peripheral blood mononuclear cells
PD: Parkinson's disease
QA: quinolinic acid
ROS: Reactive oxygen species
RLS: Restless leg syndrome
SCA: Spinocerebellar ataxia
SMA: spinal muscular atrophy
SNc: substantia nigra pars compacta
SOD1 (G93A): superoxide dismutase 1 (glycin-93 to serine)-mutant mouse
TNF-α: tumor necrosis factor alpha
TrkB: tropomyosin kinase receptor b
UTP, UDP: uridine tri-, diphosphate.
